# Radical reform of the undergraduate medical education program in a developing country: the Egyptian experience

**DOI:** 10.1186/s12909-023-04098-3

**Published:** 2023-03-03

**Authors:** Nadia Badrawi, Somaya Hosny, Lamis Ragab, Mona Ghaly, Bassem Eldeek, Ahmed F. Tawdi, Ahmed M. Makhlouf, Zeinab N. A. Said, Lamiaa Mohsen, Amira H. Waly, Yasser El-Wazir

**Affiliations:** 1 Supreme Council of Universities & Faculty of Medicine (FOM), Cairo University, Giza, Egypt; 2 FOM, Suez Canal University, Ismailia, Egypt; 3 Newgiza University (NGU), Giza, Egypt; 4 FOM, Damietta University, Damietta El-Gadeeda, Egypt; 5 FOM, Arab Council for Health Specialties, Cairo, Egypt; 6 FOM, Badr University, Cairo, Egypt; 7 FOM (for Girls), Al-Azhar University, Cairo, Egypt; 8 FOM, Zagazig University, Zagazig, Egypt

**Keywords:** Undergraduate medical Education, Curriculum, Integration – Competency based Medical Education

## Abstract

Medical educators are in a continuous quest to close the gap between the needs of medical practice and the rising expectations of the communities in their countries. During the past two decades, competency-based medical education has been evolving as an appealing strategy to close this gap. In 2017, the Egyptian medical education authorities mandated all medical schools to change their curricula to comply with revised national academic reference standards, which changed from outcome-based to competency-based. In parallel, they also changed the timeline of all medical programs for six years of studentship and one-year internship to five years and two years, respectively. This substantial reform involved the assessment of the existing situation, an awareness campaign for the proposed changes and an extensive national faculty development program. Monitoring the implementation of this substantial reform was performed through surveys, field visits and meetings with students, teaching staff and program directors. In addition to the expected challenges, the COVID-19-associated restrictions presented a significant further challenge during the implementation of this reform. This article presents the rationale for and steps of this reform, the challenges faced and how they were addressed.

**Supplementary Information:**

The online version contains supplementary material available at 10.1186/s12909-023-04098-3.

## Background

Medical education is continually reformed in response to scientific advances and societal needs [1]. During the past few decades, there have been reports of reforms from many countries around the world [2]. The common goal has been to ensure the best alignment between medical education programs and the responsibilities that graduates will face after being enrolled in national healthcare systems.

With the start of the millennium came a revolution in undergraduate medical education (UME) [2–4], which began with the introduction of a variety of approaches that promote active learning (acquiring and synthesizing knowledge through student-led activities) to complement the traditional didactic model [5, 6]. However, these reforms were pedagogical and the content reform did not expand development of the requisite skill set in future practitioners of medicine[7–9]. As a result, a worldwide mass movement aimed at producing physicians who can deliver individualized plans of healthcare occurred [5]. This requires the implementation of a spiral integrated curriculum that includes both horizontal and vertical dimensions to link it across time and disciplines [10]. This approach was emphasized by Wijnen-Meijer et al., who considered integration as a philosophy of education that influences students’ maturation and engagement with the profession and relates not only to undergraduate education but also the lifelong learning of professionals [11]. Interventions effective in developed countries may be ineffective in developing countries, which might result in different social, economic, cultural and infrastructure factors that affect how the change is implemented as well as the outcome of change.

Egypt has a long and proud tradition as a leading center of medical excellence. Many experts recognize Imhotep, of the twenty-seventh century B.C., as the world’s first physician. Moreover, Egypt is one of the first centers of medical education in the world, having established a medical school in the ninth century A.D. That tradition is being challenged by a burgeoning population and inadequate facilities to meet the demands of twenty-first century medical education. Most medical schools, public and private, exhibit problems of overcrowding and underfunding [12, 13].

While evidence of medical practice in Egypt dates back to 2,500 B.C., medical education was introduced into the university system in 1919. From that date until the turn of the millennium, few national reform initiatives occurred. Thus, until 2008, most medical schools in Egypt still adopted discipline-based curricula taught via didactic large-group lectures and apprenticeship approaches [14]. Only one medical school, since its establishment in 1978, adopted an integrated curriculum with problem-based and community-based strategies. A few other medical schools initiated a parallel track, in addition to their original traditional one, that accepts a smaller number of students where those innovative strategies have been implemented [14].

In June 2016, the planning and development committee of the medical sector, which overlooks all medical schools in Egypt, under the governance of the Ministry of Higher Education, established a subcommittee named Reform of Undergraduate Medical Program (RUMP) to develop a strategic plan for UME and to implement the developed changes. This committee performed a situation analysis of all undergraduate medical programs and determined that most of them still follow the traditional type of medical education, which is viewed as inadequate for preparing future physicians for medical practice [15, 16]. Only eight out of the 23 medical school programs at the time of the analysis made some developments towards an innovative integrated curriculum and only 8% of students were studying in those developed programs [1–18].

In 2017, the Supreme Council of Egyptian Universities (SCU) approved and enforced radical reform, mainly converting the 6-year academic and 1-year internship program into 5 years and 2 years, respectively. Additionally, the discipline-based curriculum was changed to a modular integrated model. In parallel, the National Authority for Quality Assurance and Accreditation in Education (NAQAAE) issued its second version of the National Academic Reference Standards (NARS-Medicine 2017), which was competency-based (CB) to ensure a well-integrated curriculum with early clinical exposure [19]. These standards were meant to express stakeholders’ expectations of the medical school graduate in Egypt and defined and articulated the attributes and competencies that holders of the qualification Bachelor’s degree in Medicine and Surgery should exhibit [19]. In 2017, 28 medical schools were found in Egypt, most of which were public schools.

Consequently, the RUMP committee developed a road map and prepared a general framework aligned to the 2017 NARS-Medicine to guide schools in preparation of their new integrated curricula. The developed road map was relevant to the context in Egypt and agreed upon strategies to ensure that medical education is innovative and able to prepare the undergraduates to practice in the changing scenario of medical science.

The current article presents the process followed to overhaul the UME curriculum in Egypt’ how monitoring was performed and the challenges encountered during the implementation process.

## Reform process of the medical program

**Fig. 1:**
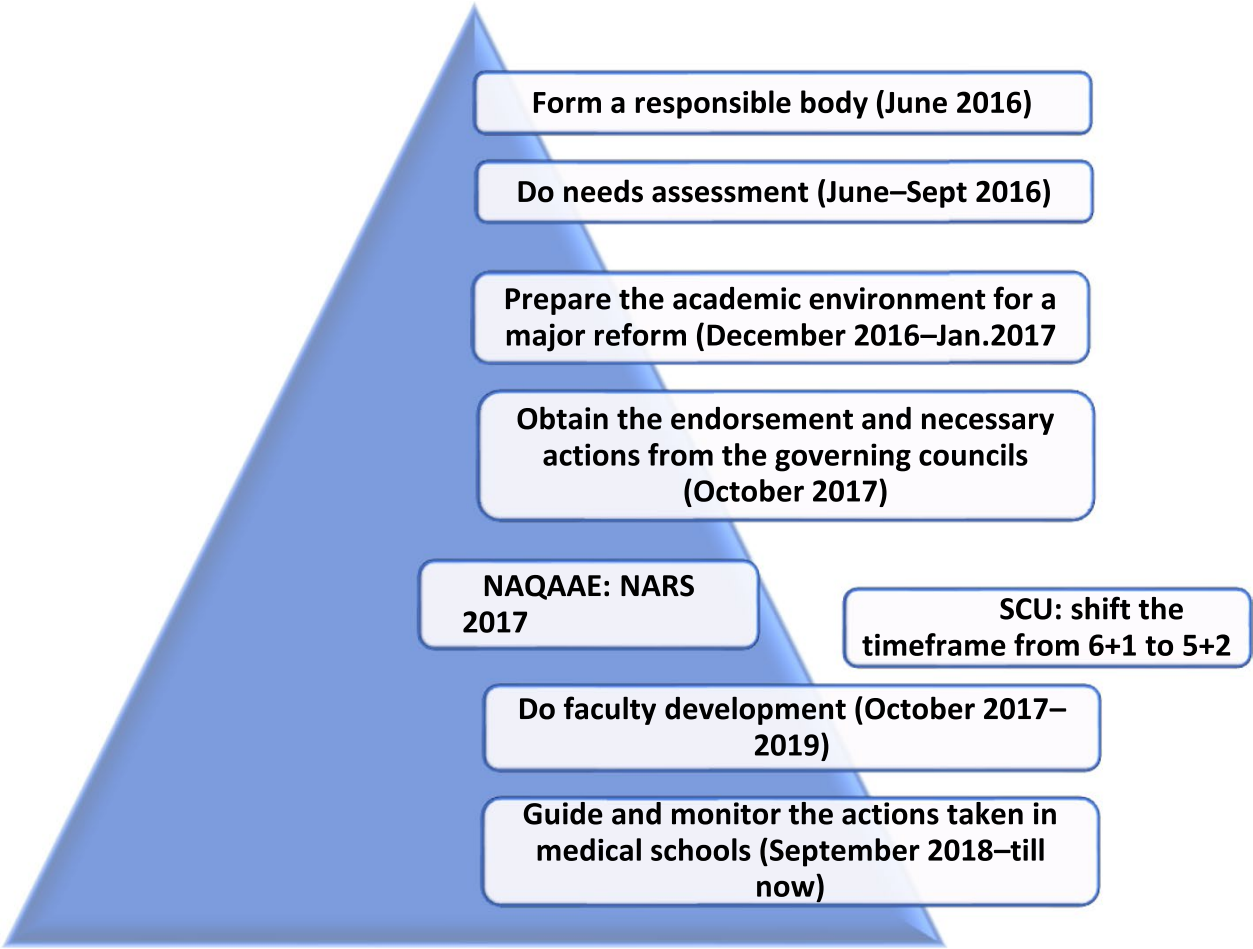
The general plan and timeline of introducing major reform in medical curricula in Egypt

To prepare and implement the intended changes in the medical schools’ curricula, the following are the actions taken and the outcomes achieved  (Fig. 1):

### Assessment of the existing situation

To assess the necessity and scope of the change, the RUMP conducted a comprehensive context evaluation, from October to December 2016, to explore the challenges of the traditional program from the stakeholders’ viewpoint. A survey was conducted to explore students’ opinions about the current situation of medical education and how to improve it (Additional file 1). The survey was prepared by the RUMP committee members and distributed to all medical schools’ students as a link on Google doc through the schools’ deans and medical student of EFMSA (Egypt Federation of Medical Students Associations). The link was available for 8 weeks. The data were saved as excel sheet then transformed to SPSS file. Revising and filtration of data were done to guard against any bias or missing data. Out of around 50,000 medical students in Egypt in 2016, 2,154 students from all academic years (one to six) participated in this initial survey. Analysis of the survey results showed that 66.6% of the students were dissatisfied with the traditional way of teaching by lectures and with the assessment methods. Additionally, knowledge recall without testing of higher levels of thinking and unfairness were the main disadvantages mentioned by the students. They also reported that they were required to resort to private tutoring in several subjects. Additionally, 70% found that their schools’ websites were not useful. These data were presented to the SCU.

A group discussion was also conducted with the academic leaders (Deans and Vice Deans of Education of medical schools) in several sessions conducted during the regular medical sector committee and attended by representatives of the RUMP committee members to get their feedback regarding the needed improvements in the existing medical curriculum. Incorporating newer teaching elements in the under-graduation course namely introduction of a foundation course after admission to prepare a student to study medicine effectively; facilitation of horizontal and vertical integration between different disciplines; advocating early clinical exposure right from the first year (viz. case scenarios for classroom discussion/case-based learning); addition of elective courses and methods to enhance self-directed learning, critical thinking and research abilities were the main responses of the academic leaders.

### Proposed changes by RUMP committee

To respond to the assessment of the existing situation results, several meetings were conducted by RUMP committee in year 2016 where different international medical curricula were reviewed in order to agree on the proposed curriculum changes. The committee reached a consensus to formulate and adopt a competency based medical education national framework. The committee also recommended effective monitoring of the compliance of medical schools in implementing horizontal and vertical integration, self-directed learning, early clinical exposure, more emphasis on clinical practice and implementation of credit hour/point system.

### Awareness campaign for all medical schools

To orient the medical schools about the intended changes in the medical program, an awareness campaign started in December 2016 and ended in January 2017. All governmental and private medical schools all over Egypt at that time were visited. There were 28 medical schools at that time, the public schools constituting the majority. Each visit was conducted by 2–3 RUMP members. The awareness package included information about the rationale for the change to competency-based medical education (CBME), the relevance to NARS-Medicine 2017, the definition, importance, types and levels of integration. Different models of curricula from UK, Europe and Arab countries were discussed as well**.**

The awareness package was delivered to the stakeholders in each school including students, recent graduates, academic leaders and faculty members. At least 40% of students and 30% of faculty members attended each awareness campaigns.

### Preparing the framework for reform and getting higher authorities’ approvals

A series of meetings and focus group discussions were made between the coordinator of the RUMP, the head of the Medical Sector Committee (MSC) and the secretary general of the Supreme Council of Universities (SCU) and the relevant ministers in the period from June to September 2017 to propose the framework of reform and the required changes in the laws and bylaws. The required changes were approved by the Ministries’ Council on the 4^th^ of October/2017 including modification of the bylaws clause 154, so that the period of undergraduate studies for the medical school in Egypt will be 5 years competency- based integrated program using a credit system followed by two years of internship and a licensing exam before practice. The SCU approved that the new program should be implemented by all medical schools starting from the academic year 2018/2019.

### Preparing curriculum maps and bylaws by all medical schools

After getting the higher authorities’ approvals, two documents stating a framework for preparing the bylaws and curriculum maps were prepared by the RUMP committee and issued to all schools from the SCU to follow in preparing their curricular maps and new bylaws. Continuous communication occurred between RUMP members and the curriculum committees in each medical school during its work on bylaws and map to be ready for the approval of the new reform. In April to June 2018, RUMP members revised each medical school curriculum map and bylaws according to a pre-set standard checklist to ensure that each program fulfils the updated NARS-Medicine, 2017 requirements and the items stated in a document issued from SCU concerning the framework of the bylaws. RUMP members feedback was given to the schools and modifications were done accordingly. Before the start of the academic year 2018/2019 the bylaws of all medical schools were approved by the SCU which is the higher authority.

The framework of the new bylaws covered all the points that should be included in the bylaw such as: information about the College and University, graduate attributes, vision, mission, strategic objectives, educational strategies, departments and committees, admission process, the duration of study, the program structure, credit hours or credit points data and many other important instructions regarding complaints, withdrawal, transfer, etc. The bylaw also defined certain important items that should be emphasized in the curricula which are: 1) description of the integrated modules including types of integration and its level in addition to the used teaching methods that can achieve this integration. 2) the presence of a preparatory period for the students to be oriented with the new program and the presence of a study guide. 3) Self-directed learning and ways of maximizing it. 4) Early clinical exposure. 5) Communication and professionalism and other skills that are mandated in the NARS competencies. 6) Electives 7) Research methodology and research skills to be included in the early years of the program. 8) Including formative and summative assessment in addition to using new objective methods of student assessment that measures higher cognitive domain and practical and professional skills. 9) implementing quality assurance measures on the program.

The main curricula changes done by the medical schools in their new curricula were introducing different levels of integration according to the schools’ infrastructures and personal competencies, but all were exceeding level 5 according to Harden ladder [20]. Modern teaching and learning methods were used, mostly interactive and self-directed. Almost all the schools added early clinical teaching mainly by using clinical skills labs and early experience with patients in different clinical settings. Most of the schools introduced an introductory module, in year one, preparing students for further undergraduate studies. All schools added elective module(s). Research methodology was introduced into all curricula. Assessment policies witnessed a dramatic change, getting away from knowledge recall and using objective, structured methods.

### Capacity building

In preparation for implementation, a faculty development program under the title of teaching excellence in medical education was conducted by the Egyptian Knowledge Bank (EKB) in collaboration with the Medical Military Academy (Additional files 2 and 3). Throughout the years 2017—2020, the EKB organized 48 workshops of 430 h in total. The workshops were facilitated by 13 expert educational leaders from the United States of America, United Kingdom, Australia and Canada and were attended by around 300 participants. The main areas of training were excellence in curriculum design, teaching & learning and assessment. This faculty development program started in 2017, before the actual implementation of the new curriculum, with eight workshops and continued in 2018, after implementation, with ten workshops, one special service workshop for deans and one seminar conference. In 2019, 22 workshops and one seminar were conducted. In 2020, five workshops were done. In parallel, the EKB also provided free access to digital training resources, such as Incision Academy and Virtual Patient Learning to the medical students at all medical schools. All medical schools benefited from EKB services and 81,086 students logged in to the Incision Academy portal. The conducted workshops were considered a training of trainers (TOT) for continuing training of all involved faculty in all schools. In addition to this TOT, each medical school started its own faculty capacity building with the support of RUMP members. The evaluation conducted by EKB for the faculty development program in 2019 revealed that the responses of participants were as following: 97% stated that the courses met their expectations, 87% affirmed that they would recommend the courses to their colleagues, 87% said that they believe their professional work will improve as a result of the courses and 88% rated the course leader’s knowledge as above average/outstanding. The evaluation conducted by the EKB for the year 2021’s courses showed that the total satisfaction rate of the participants was 96%. The responses were: we gained valuable new ideas or insights from the attended workshops (97.5%), we gained practical knowledge after attending these workshops (95%) and we will share what we learned during these workshops with our colleagues (98%).

### Starting implementation

The new curriculum model implementation started on September 2018, for the first year students. This included horizontal and vertical integration of content, introduction of an orientation module, stronger clinical orientation starting already in the first semester, more elective components and integrated examinations. The curriculum included the modular line components (mostly organ/function modules lasting 3–6 weeks) supplemented with semester-vertical courses. Each medical school selected the coordinators for all modules of the new curriculum after an application process. These coordinators had the responsibility of choosing an interdisciplinary planning team of at least six members, including faculty members from non-clinical as well as clinical subjects. These planning teams were charged with selecting the content which would be covered in their module as well as the appropriate learning methods.

### Monitoring (audit) visits

The RUMP members designed a comprehensive, multicomponent and program-wide monitoring system. The framework expects the quality of the program as comprising four main aspects: curriculum and resources; staff and teaching; student experience; and management support. Key principles of the adopted audit included feedback form students and staff, auditing of teaching, learning and assessments methods. An emphasis on corrective actions following evaluation was included (closing the loop), which was undertaken by teachers, course coordinators, and administrators. An external auditing checklist was prepared by the RUMP committee to be filled by the assigned auditors. The checklist included readiness of the bylaws, program specifications, course specifications, the curriculum matrix, the action plan, teaching and learning methods (interactive sessions, problem-based learning (PBL), self-directed learning (SDL), simulations, field visits and practical/clinical training), as well as details of the assessment process (strategy, blueprint, formative assessment documents and summative assessment tools).

Four meetings were held with the auditors who include the RUMP committee members in addition to a selected group of faculty members from different universities who have qualifications and experience in medical education. One meeting was held at the beginning of the academic year 2018/2019 to standardize the auditing process and discuss the items of the external auditing checklist with the auditors. The other meetings were held before each cycle of monitoring visits. Three in-person monitoring visits were conducted: two in the first year of implementation (once per semester) and the third one in the first term of the academic year 2020/2021. The third visit occurred during the evolving COVID-19 pandemic. Because of the COVID-19 lockdown, auditing in the academic year 2020/2021 was paused. In each auditing visit, two assigned RUMP members visited each school. The auditing visits in 2021 were mostly done online.

The filled checklists were included in a final report. Generally, the auditors found that the program specifications were prepared in almost all the faculties by the 2020 audit. Conversely, the alignment matrix between the 2017 NARS-Medicine and program intended learning outcomes (ILOs) were completed in about three-quarters of the faculties visited. The reform action plans that were formulated by the faculties to address the comments of the audit reviewers were implemented in 71% of faculties in the first audit and 80% in the second round of the audit.

### Students’ and faculty feedback after implementation

To assess the students’ and faculty’s points of view after implementation, questionnaires, designed by RUMP committee, was distributed to all medical schools through a survey monkey application. The first student survey (Additional file 4), conducted in 2019, received 9,044 responses. Students reported that the preparatory period and study guides were beneficial (70%), the interactive teaching methods were interesting (50%), the teachers spent efforts on their training (50%) and they used the schools’ websites (40%). The second student survey (Additional file 5), conducted in the year 2020, received a total of 18,655 responses. The students’ main responses were that the learning outcomes were well explained (60%); the websites were useful (60%); they received training in research and clinical skills (40%); they had courses in communication and ethics (50%); and electives were applied (40%). Fifty percent of the students were satisfied by small-group teaching, clinical teaching and formative and continuous assessment (50%). Additionally, 40% of the students expressed their satisfaction regarding E-learning and the received feedback.

Regarding feedback of the faculty members, their implementation survey (Additional file 6) elicited 1,911 responses. About 95% stated that a team exists for implementation of the integrated medical program in their faculties. Half of the respondents indicated their involvement in the implementation of the program. About 70% of the respondents received training either through the EKB or in their faculty about the concept of integration, its design and implementation. Only 60% received online training for teaching and assessment and 70% of the respondents stated that their students received teaching in small groups.

### Coping with the pandemic

In order to manage the teaching and learning activities during the COVID-19 pandemic, all the faculties adopted a blended learning approach of online and on-campus activities. In the auditing visits, auditors noticed that most of the faculties used the Microsoft Teams platform as well as the EKB resources. The utilized EKB resources included the Incision Academy and Virtual Patient Learning. Although free access to the EKB learning management system was provided, some faculties implemented the open-source learning platform MOODLE (Moodle Pty Ltd, West Perth, WA, Australia) and continued to utilize its options during the pandemic.

Conversely, variable approaches occurred for the assessment process during the pandemic. Most faculties used online formative assessments to motivate the students learning. Regarding final written tests, some faculties used online assessments, while other faculties requested that students submit review articles that were uploaded online. Considering the practical skills, they were assessed by objective structured practical exam (OSPE) on campus with the application of all COVID-19 prophylactic procedures.

### Challenges of implementation reported by auditors

During the implementation of the new medical curriculum, RUMP explored the challenges by questioning the auditors who met representatives of faculty, students and leaders of medical schools during the monitoring visits. Their answers can be summarized in the following points:

- Skepticism and resistance were found from some faculty members who felt that such young students would be unable to adopt self-directed learning. Faculty members of certain disciplines expressed concerns that the new scoring system in the integrated curriculum, which assigned scores to learning outcomes rather than disciplines, would encourage some students to ignore subjects that contributed a small share in some modules. Because the integrated modules followed a common sequence, some faculty members argued that this order was the most suitable one for their disciplines. Some believed that moving the students into the modular system would not provide a good base of the required knowledge in all disciplines. Others saw that the integrated system required that instructors must change the way of teaching, which they had been adopting for a long time. Collaboration with colleagues from other departments would also be needed to plan classes. In other words, they would be required to make an extra effort that they used to do in the original curriculum.
- Many students complained of marked overload in the content of some modules, apparently because some departments did not do enough effort to tailor the content to the shortened time of their disciplines.
- Early clinical exposure could not be satisfactorily achieved in some medical schools because of the large number of students and logistic difficulties in securing the required training places.
- Some RUMP reviewers reported that in some medical schools, the level of horizontal integration was limited to synchronization without actual exchange of material and educational plans to ensure proper sequencing of educational activities.
- In some schools, examinations, especially in the first year, largely addressed recall with a small percentage of questions devoted to testing higher cognitive levels.
- Lastly, the COVID-19 pandemic presented an extra burden during the implementation.

## Discussion

In 2016—2018, the governing councils of medical education in Egypt managed to introduce major nation-wide reform in medical curricula. This was done mainly in response to the feedback of medical students and academic leaders about the effectiveness of existing medical curriculum at that time. The expectations of the Egyptian community regarding the quality of medical graduates and the current trends in medical education were also other important driving factors.

Introducing such a significant change in the structure and timeline of the medical curriculum, at a national level, is quite challenging and could stumble at any stage. Actually, there was a previous trial of major reform in medical education and practice in Egypt several years ago that did not make it because of much resistance from different parties. Resistance to substantial change in higher education institutions is a frequent encounter despite that these institutes should initiate change in their societies [21]. Because of the expected resistance and the previous incomplete attempt, RUMP committee decided to present a clear and well- articulated plan from the very beginning.

The most important strategy throughout the whole process was to involve the essential stakeholders in every step, especially, the students, teaching staff and decision-makers to convince them about the change. This coincides with the Lewin’s three-step model of change management [22, 23]. This simple model starts by the first phase which is challenging core beliefs of the people working in the organization. The second phase is presenting the alternative and getting the needed support. The third phase is implementing the change. It is well known that the first phase of Lewin’s three-step model presents the greatest challenges as people feel uncertain and lost [23]. Thus, RUMP committee spent a lot of effort to prepare the academic medical community for this substantial change, through visiting all medical schools in Egypt in December 2016 and January 2017 and attended meetings with the program leaders, teaching staff and students.

Exploring the students’ opinions regarding their medical curriculum, before implementing the new program, showed a high level of dissatisfaction (66.6%) due to the high load of theoretical knowledge and the reduced time devoted for skills acquisition. An alarming point, in the narrative part of the questionnaire, was the extent of the problem of private tutoring. Many students reported their need of private tutoring to compensate for some issues in their program, such as a relatively deficient clinical training. In addition to collecting students’ feedback, focus group discussions with the academic leaders in all medical schools also corroborated the main findings of the students’ survey.

In response to these important comments and to narrow the gap between medical education and real practice and ensure a better transition from theory to practice, the governing councils adopted the CBME framework which emphasizes the professional skills in the curriculum. To serve the same purpose, 1 year was moved from studentship phase to the internship phase, so that the timeframe became 5 years plus 2 years instead of 6 years plus 1 year. This curriculum duration is consistent with UK General Medical Council (GMC) requirements.

Another motive for adopting CBME is the global trend in this direction. During the last two decades, the national medical authorities in several countries, such as Canada [24], USA [25], Netherlands [26], Saudi Arabia [27] and India [28] shifted their postgraduate and/or undergraduate medical curricula to the competency-based paradigm. The implementation process in each country followed certain steps that shared the following steps: needs assessment, planning, obtaining official approval, implementation, evaluation and follow-up. Additionally, implementing CBME has been a recommendation of some national and regional experts in medical education. During the last years, the recommendations of professional regional meetings of medical education experts were in accordance of adopting CBME. In a WHO EMRO workshop in November 2014, curriculum development of medical programs was identified as a key area for development [29]. Proposed specific priority actions include agreeing on a regional set of competencies including professionalism, communication skills, ethics, legal medicine, as well as encouraging early community and patient contact of medical students to gain appropriate professional competencies and clinical skills and extending the clinical training to all health care facilities [30].

Egypt was no exception in this regard, as it decided in 2016 to start the update of the first version of NARS, issued by NAQAAE in 2009, to be a competency based one. This first version of NARS 2009 was an outcome-based in which the outcomes were categorized as knowledge, cognitive skills, professional skills and general skills. During application, it was obvious that this categorization is rather artificial. Therefore, the second version of NARS-Medicine which was issued in 2017, adopted the competency-based framework [19] with consequent mandates of early clinical exposure, horizontal and vertical integration and frequent formative assessment.

Before implementation of the new curriculum, each medical school was required to go through a mandatory regulatory process to update its bylaws. A general framework of the new bylaws was prepared by the RUMP as a guide and each medical school could modify it according to its own context because medical schools in Egypt vary in terms of the resources, intake capacity and number of the teaching faculty.

The awareness campaign at the start of the implementation process was important to create readiness for the reform in the academic environment.

Before this curriculum reform, only a few medical schools in Egypt were implementing active student-centered strategies and the premise of the CBME learner-centered approach was unfamiliar. Most programs were mainly relying on the lectures and practical sessions as the principal instructional methods. Since CBME is a learner centered approach, the RUMP emphasized proper implementation of active teaching methods in all medical schools. Thus, all programs introduced small group tutorials, flipped classrooms, students field projects, e-courses or team-based learning from year 1. Previous data of implementing CBME in undergraduate curriculum stated the key role of active learning in this kind of curriculum [31].

In parallel to the change in instructional methods, all medical schools started implementing reform in tools of students’ assessment. Specifically, all schools were required to use portfolios for monitoring students’ progress and to provide training in self-evaluation and reflection. Some programs replaced the printed portfolios first used with an electronic one to facilitate the whole process. This action was essential in the successful implementation of the CBME model, given the key role of continuous assessment and frequent feedback in the development of the planned competencies. Additionally, it improved student-staff communication and facilitated the monitoring of competencies acquisition via different activities and assessment methods. This finding was supported by other studies [32].

One of the important strategies during implementation is organizing an extensive training and support to the faculty. It has been reported that faculty development is one of the strategies to face the resistance to change during the transition to CBME [33]. Thus, faculty development program was organized for the teaching staff on many topics related to CBME and other relevant topics and the evaluation conducted by EKB as presented in the previous section showed satisfactory results.

Additionally, the feedback obtained from the faculty and the students after implementation revealed some strengths such as the relatively large number of faculty who received training and the satisfactory level of students’ awareness of the learning outcomes. Points that need more emphasis in some schools’ curricula were the research, clinical skills and elective modules. Programs are alerted of areas of concern and corrective plans were to be followed-up during future monitoring visits, which will continue on annual or semiannual basis according to the needs of each school.

Evidence exists from the literature that extending the duration of well-designed internship training improves acquisition of clinical skills [34, 35]. The internship period is important in the Egyptian context because many Egyptian graduates continue their career as general practitioners and do not enroll in postgraduate programs. Thus, extending the duration and improving the quality of the internship program is expected to result in a positive impact on the quality of health services offered by those general physicians to the Egyptian community. The perception of interns of the impact of internship training in building their independent professional practice has been reported differently in different countries [36, 37]. It is obviously dependent on the quality of the training offered during the internship program. While being generally favorable in some areas [36], it was criticized in some other countries [37]. Although this part has not yet started, RUMP is keen to design it as a continuation of the undergraduate competency-based framework, with well-defined competencies, milestones and entrustable professional activities (EPAs). This design can be guided by some recently proposed models [38].

## Conclusion

A radical reform was made in the medical education program in Egypt in collaboration with different stakeholders. The reform was done in response to the new advances in medical education and the competency-based framework. Regular monitoring and evaluation of the new implemented program occurs. However, thorough evaluation should be performed after the graduation of the first batch in 2023 to receive an overall idea about the challenges of the implementation and to develop an action plan for improvements.

## Supplementary Information

**Additional file 1.** Needs assessment questionnaire 2016-2017.

**Additional file 2.** Teaching excellence in Egyptian medical education.

**Additional file 3.** Teaching excellence in medical education assessment design.

**Additional file 4.** Student survey 2018-2019.

**Additional file 5.** Student survey 2019-2020.

**Additional file 6.** Faculty perceptions and practices in the new (Integrated) program questionnaire.

## Data Availability

Data and materials are available by contacting the corresponding author.

## Abbreviations

UME: Undergraduate medical education
RUMP: Reform of Undergraduate Medical Program
SCU: Supreme Council of Egyptian Universities
NAQAAE: National Authority for Quality Assurance and Accreditation in Education
NARS: National Academic Reference Standards
CBME: Competency-based medical education
MSC: Medical Sector Committee
EKB: Egyptian Knowledge Bank
