# Supplementary material for: Radical reform of the undergraduate medical education program in a developing country: the Egyptian experience
Source: BMC Med Educ. 2023 Mar 3;23:143. doi: 10.1186/s12909-023-04098-3 (PMC9983512; doi:10.1186/s12909-023-04098-3)
Supplement: Supplementary file 1 — Additional file 1. Needs assessment questionnaire 2016-2017. [file 12909_2023_4098_MOESM1_ESM.pdf]

Annex 1

Needs assessment questionnaire 2016-2017

**Faculty**	
**year of the study**	
**GPA**	

	1.Stongly agree	2.agree	3. Neutral	4. Disagree	5. Extremely disagree
1. Methods of teaching are effective and attractive to the students.					
2. Teaching linked to clinical cases .					
3. Correlation between theme/topic and discipline is implemented in the curriculum.					
4. Theoretical exams are fair and discriminating between the students					
5. clinical exams are fair and discriminating between the students					
6. Practical exams are fair and discriminating between the students					
7. Oral exams (if applicable) are fair and discriminating between the students					
8. Exams have effect on improvement of professional and educational levels					
9. Study of medicine helps in learning communication skills					
10. Study of medicine helps in learning research skills					
11. Study medicine in basic years helps to study in clinical year.					
12. Study medicine helps to prepare student to international certificate.					
13. Student feedback is regular about teaching and learning					
14. Students have the chance to know the results and effect of feedback.					
15. Courses outside the faculty plays important role in medical education.					
16. If the faculty offer good medical education, the students do not have to go for these courses					
