# Supplementary material for: Radical reform of the undergraduate medical education program in a developing country: the Egyptian experience
Source: BMC Med Educ. 2023 Mar 3;23:143. doi: 10.1186/s12909-023-04098-3 (PMC9983512; doi:10.1186/s12909-023-04098-3)
Supplement: Supplementary file 2 — Additional file 2. Teaching excellence in Egyptian medical education. [file 12909_2023_4098_MOESM2_ESM.pdf]

Egyptian Knowledge Bank
بنك المعرفة المصري

TEACHING EXCELLENCE IN EGYPTIAN MEDICAL EDUCATION

PROGRAM OVERVIEW

Knowledge E
Engaging minds

Background

Knowledge E is pleased to present the following comprehensive, capacity-building training program aimed for the advancement of teaching excellence application within medical education in Egypt. After successfully completing a pilot program in 2017, Knowledge E has been tasked to improve upon and expand the program for 2018.

Moreover, the updated program will aim to aid in the skill development and capacity building of key participants that will be vital to the execution of the national goals to move towards a fully integrated, competency-based, 5+2 structed medical education curriculum. Detailed below is the initial proposal for this initiative.

Program Overview

This comprehensive course will focus on delivering **practical skills** that will foster **application** of **international best practices** related to teaching excellence in medical education.

General Program Aims

1. Participants will be familiarized to the use of parameters and guidelines for teaching practices and excellence attributes in learner-centered learning environments.
2. Participants will be actively engaged throughout the exploration of the creative, critical thinking, and problem-solving processes by using teaching tools and systems.
3. Participants will be empowered to employ tools and systems for maximizing learning objective outputs and measurements through strategically integrated curriculum and assessment design.
4. Successful participants will become fluent users of practical tools and resources for teaching methods that engage learners, foster growth, and achieve lasting learning outcomes.

Pedagogical Overview

The teaching methods for this course will correlate with competency-based assessment and will focus on achieving practical learning outcomes by engaging learners and delivering tangible results. The interactive workshops will combine expert content delivery with hands-on exercises and practical exercises to maximize retention and empower learners to achieve success beyond the classroom.

Delivery and Duration

The program will focus on delivering 4 learning modules based on core-competencies (as outlined below) that have been identified as the most relevant and useful competencies for the learners based on the most recent medical educational mandates of the Egyptian government, key Egyptian stakeholder feedback, and international expert consultation.

The program will specifically cater to the needs of the learners by allowing extended time on the necessary topics. The program will be delivered to two separate cohorts of learners (cohort A and cohort B). The learning modules will be completed across a total duration of 16 days (96 hours) per cohort and will be delivered between April– December 2018.

© 2018 Knowledge E. All rights reserved. This document is strictly confidential and not legally binding.

Core Competencies

With the aim of achieving a graduate profile based on competencies, the following learning modules have been designed in keeping with feedback from key stakeholders and expert inputs. (See ANNEX A).

1. **Theory Guided Teaching and Learning**
   a. Introduction to teaching and learning
   b. Overview of adult learning theories
   c. Overview of competency-based learning models
   d. The value of implementing student-centered teaching models
   e. Practical tools for fostering and promoting andragogy in your institution
      i. Small group/ large group teaching
      ii. Clinical teaching at the bedside
      iii. Student mentoring
   f. Practical tools for creating peer networks for sharing best practices

2. **Applied Curriculum and Course Design**
   a. Implications of theory on course design
   b. Foundations of competency-based education
   c. Foundations of integrated curriculum design
   d. Fostering an integrated curriculum through organizational structure
   e. Practical tools syllabus blueprinting and session planning
   f. Course evaluation and quality assurance systems
   g. Curriculum analysis
   h. Transitioning techniques for successfully implementing curriculum reform

3. **Assessment Design**
   a. Foundations of competency-based assessment
   b. Best practices for question design and implementation (building the Egyptian question bank)
   c. Practical tools of assessment blueprinting
   d. Practical tools for OSCE and OSPE design
   e. Techniques for assessing soft skills
   f. Workplace based assessment
   g. Uses of portfolio assessment
   h. Quality assurance for assessment
   i. Creating a sustainable culture of self-evaluation, professional community development, and quality assurance (as related to assessment)

4. **Practical Application of Teaching Excellence Skills (Cumulative)**
   a. Summative group activities and exercises that will foster the practical (real world) implementation of the key learning outcomes of the previous workshop units. Each activity will:
      i. Correspond to and expand upon the previous units' individual assessments
      ii. Foster application at the participant's home institution and peer collaboration
      iii. Allow for meaningful participant- specific feedback

© 2018 Knowledge E. All rights reserved. This document is strictly confidential and not legally binding.

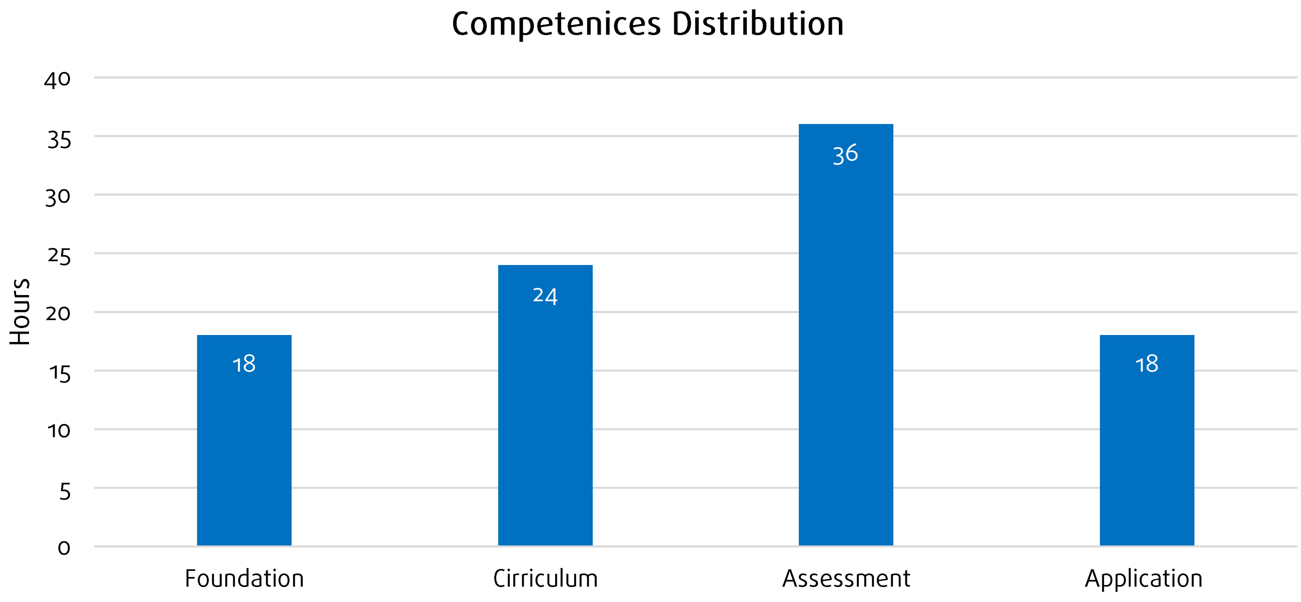

Program Requirements

In order to successfully complete the program and receive the relevant certificate, participants must meet the following requirements:

a. Overall course attendance of 87.5% or higher. This means that a participant may be absent no more than 2 days across the entire duration of the program.
    a. Please note that if a participant arrives at a workshop more than 1 hour late, their attendance will NOT be counted for that day.
b. Participation in all workshop exercises, engagement with the course leaders, and interaction with peers as relevant for each module.
c. Completion of all pre-course reading and preparatory assignments.
d. Satisfactory completion of all course assignments including the 3 formative assessments that will be administered at the end of each content module AND the summative assessment that will administered during the completion of the final workshop.

Participant Selection

Course Capacity

No more than 35 learners will be accepted per cohort. Each Egyptian medical school will be represented.

Application Process

Phase 1: Submission of online application
Phase 2: Review of application by selection committee
Phase 3: Finalization of approved participants and formal commitment from learners

Desired Participant Attributes

a. Passionate display of growth mindset
b. Proof of capacity to spread knowledge obtained (role within their institution)
c. Fluency in English (spoken and academic writing)
d. Commitment to complete all program requirements

© 2018 Knowledge E. All rights reserved. This document is strictly confidential and not legally binding.

Participant Assessment

To ensure the achievement of the intended learning outcomes, formal assessments will be utilized to evaluate the participants. This assessment will be;

1. Designed by the expert program supervisor to ensure international best practice in assessment design and correlation to desired learning outcomes for each module.
2. Elevated by the course leader and the expert program supervisor to ensure non-bias and equitable marking.
3. Formative
    a. Individual assessments will be administered at the end of each module related to the core competencies of each topic.
4. Summative
    a. An additional summative assessment will be given at the end of the program to ensure participant's cumulative comprehension and content synthesis.

Blended Learning

We aim to enhance the delivery of the program by utilizing a combination of onsite and online modules within the EKB framework, similar to other internationally leading medical education programs to support;

1. Pre-workshop reading and assignments
2. Workshop content including PPTs, workbooks, videos, and complementary materials
3. Post-workshop assignments

Expert Program Supervision

In addition to providing international experts as leaders for each individual workshop, we propose the engagement of a top international professional to provide comprehensive strategic input for the successful execution of the program. The expert's key roles shall include;

1. Review and recommendation of individual workshop leaders
2. Review and recommendation of course content
3. Design of the participant assessment
4. Evaluation of the participant assessment

© 2018 Knowledge E. All rights reserved. This document is strictly confidential and not legally binding.

Program Schedule

Cohort	Module	Course Leader	Date	Duration
A	Theory Guided Teaching and Learning	Prof. Hossam Hamdy	April 28-30	3 days (18 hours)
A	Applied Curriculum and Course Design	Prof. David Taylor	June 21-23	4 days (24 hours)
A	Assessment Design (Part 1)	Prof. Adrian Freeman	October 7-9	3 days (18 hours)
A	Assessment Design (Part 2)	Prof. Maryellen Gusic	November 11-13	3 days (18 hours)
A	Practical Application of Teaching Excellence Skills	Prof. Michelle McLean	December 15-17	3 days (18 hours)
			TOTAL (Cohort A)	**16 days (96 hours)**

Cohort	Module	Course Leader	Date	Duration
B	Theory Guided Teaching and Learning	Prof. Hossam Hamdy	May 2-4	3 days (18 hours)
B	Applied Curriculum and Course Design	Prof. David Taylor	June 25-27	4 days (24 hours)
B	Assessment Design (Part 1)	Prof. Adrian Freeman	October 11-13	3 days (18 hours)
B	Assessment Design (Part 2)	Prof. Maryellen Gusic	November 15-17	3 days (18 hours)
B	Practical Application of Teaching Excellence Skills	Prof. Michelle McLean	December 18-20	3 days (18 hours)
			TOTAL (Cohort B)	**16 days (96 hours)**

© 2018 Knowledge E. All rights reserved. This document is strictly confidential and not legally binding.

Knowledge E
Engaging minds

ANNEX A
CORE COMPETENCIES MATRIX

Theory Guided Teaching and Learning

- Introduction to teaching and learning
- Overview of adult learning theories
- Overview of competency-based learning models
- The value of implementing student-centered teaching models
- Practical tools for fostering and promoting andragogy in your institution
  - Small group teaching
  - Large group teaching
  - Clinical teaching at the bedside
  - Student mentoring
- Practical tools for creating peer networks for sharing best practices

Applied Curriculum

Applied Curriculm and Course Design

- Implications of theory on course design
- Foundations of competency-based education
- Foundations of integrated curriculum design
- Fostering an integrated curriculum through organizational structure
- Practical tools syllabus blueprinting and session planning
- Course evaluation and quality assurance systems
- Curriculum analysis
- Transitioning techniques for successfully implementing curriculum reform

Assessment Design

- Foundations of competency-based assessment
- Best practices for question design and implementation (building the Egyptian question bank)
- Practical tools of assessment blueprinting
- Practical tools for OSCE and OSPE design
- Techniques for assessing soft skills
- Workplace based assessment
- Uses of portfolio assessment
- Quality assurance for assessment
- Creating a sustainable culture of self-evaluation, professional community development, and quality assurance (as related to assessment)

Practical Application of Teaching Excellence Skills (Cumulative)

- Summative group activities and exercises that will foster the practical (real world) implementation of the key learning outcomes of the previous workshop units. Each activity will:
  - Correspond to and expand upon the previous units' individual assessments
  - Foster application at the participant's home institution and peer collaboration
  - Allow for meaningful participant- specific feedback

© 2018 Knowledge E. All rights reserved. This document is strictly confidential and not legally binding.
