# Supplementary material for: Radical reform of the undergraduate medical education program in a developing country: the Egyptian experience
Source: BMC Med Educ. 2023 Mar 3;23:143. doi: 10.1186/s12909-023-04098-3 (PMC9983512; doi:10.1186/s12909-023-04098-3)
Supplement: Supplementary file 3 — Additional file 3. Teaching excellence in medical education assessment design. [file 12909_2023_4098_MOESM3_ESM.pdf]

Knowledge E

Engaging minds

Presents

Teaching Excellence in Medical Education

Assessment Design

Egyptian Knowledge Bank

بنك المعرفة المصري

About the Egyptian Knowledge Bank

The Egyptian Knowledge Bank (EKB) is a national initiative to simulate learning throughout the nation by providing Egyptian's with the tools, content and training the require to build a knowledge economy based upon the principles of learning for everyone.

An integral part of the EKB initiative is a nation-wide training programme that seeks to reach people from Egyptian universities, research institutes, publishers, hospitals, ministries, academies and any other place dedicated to the advancement of learning and knowledge creation in higher education.

For more information about the EKB visit:

Website: www.ekb.eg
Facebook: www.facebook.com/EgyptianKnowledgeBank
Twitter: twitter.com/EgyKB

About Knowledge E

Established in 2012, Knowledge E is an educational services and products provider based in Dubai, UAE. Our vison is 'a more knowledge world' and we believe that every academic professional and educational institution deserves the opportunity to excel, whether in a niche area, or on the global stage.

Knowledge E provides scholarly content to academic institutions throughout the Middle East, and provides training courses to researchers, scholarly editors, librarians, university leaders, and students. Combined with publishing and editing services, we aim to provide knowledge to research communities in emerging regions and enable them to globalise their own results, thereby deepening integration in the international knowledge network. Visit www.knowledgee.com for more information on any of our services:

Capacity-Building Training | Publishing Journals & Conference Proceedings | Digital Library

© 2017 Knowledge E. All rights reserved. This workbook is intended for non-commercial use and may not be reproduced, distributed, or transmitted without the prior written permission of the publisher.

Table of Contents

Workbook Author 2
About the Egyptian Knowledge Bank 2
About Knowledge E 2
Course Overview 4
Course Leader 5
Overview of Assessment, Validity & Reliability 6
Effective assessment methods 11
Interactive session: writing high quality assessment items 22
Assessment blueprinting – aligning & integrating with the curriculum 24
Standardising Assessment: formative and summative assessments 29
Interactive session: Standard setting the borderline/pass mark 35
Workshop: Using SCT to assess and enhance clinical reasoning 39
Evaluation of student performance in assessment 48
Clinical and performance based assessment 48
Giving feedback to students to ensure lasting knowledge 56
Developing and running OSCE – common pitfalls 60
Challenges in assessment 68
Wrapping up- future collaborations 72

© 2017 Knowledge E. All rights reserved. This workbook is intended for non-commercial use and may not be reproduced, distributed, or transmitted without the prior written permission of the publisher.

Course Overview

Course Summary

This workshop will aim to guide participants on effective assessment design, allowing them to ensure lasting knowledge for their students. In this workshop, you will explore the core components of effective assessment and practice assessment design.

This course will provide a comprehensive review of the important components in Assessment Design. A series of short lectures on core concepts in assessment will be followed by interactive hands-on sessions to engage participants in developing high quality assessment items to assess clinical reasoning, standard setting and giving constructive feedback to students. This course will aim to train participants on:

- Understanding common effective assessment methods
- Developing high quality assessment items to assess students and enhance learning
- Standard setting of assessment
- Using Script Concordance Testing to assess and enhance clinical reasoning
- How to give effective feedback to ensure lasting knowledge
- Providing effective Clinical and performance based assessment
- Understanding the current challenges in assessment

Course Introduction

As all educators know, assessment is the cornerstone for measuring student learning outcomes. When meaningfully used, assessments can be an outstanding tool for implementing true teaching excellence with our students.

In this course, we will overview the elements of meaningful and effective assessments by exploring validity and reliability, blueprinting, curriculum alignment, and assessment standards. We will apply this knowledge through hands on exercises for writing high quality assessment items that utilize formative and summative assessments to achieve lasting knowledge for students. Thus, allowing for more efficient and accurate evaluation of student performance through our assessments.

We will also utilize practical tools and resources that will help to strengthen your capacity for excellent assessment deign, create strategies for overcoming challenges in assessment design, and foster opportunities for future collaborations with our fellow medical educators.

© 2017 Knowledge E. All rights reserved. This workbook is intended for non-commercial use and may not be reproduced, distributed, or transmitted without the prior written permission of the publisher.

Overview of Assessment, Validity & Reliability

Getting started:

What do you think is the main purposes of Assessment?	
What is formative assessment?	
What is summative assessment?	
What types of assessment do you use in the School?<br>e.g. MCQ, EMQ, OSCE	
How do you set the pass mark of your various assessments?	
How do you assess clinical reasoning?	
Have you heard of Script Concordance Test (SCT) for clinical reasoning?	
Are you aware of any national or international benchmarking exercises in Assessment?	

© 2017 Knowledge E. All rights reserved. This workbook is intended for non-commercial use and may not be reproduced, distributed, or transmitted without the prior written permission of the publisher.

What is assessment?

"Assessment is the process of forming a judgement about the quality and extent of the performance of a student."

(Prof Royce Sadler, 2007)

"An ongoing process of gathering and interpreting information about a learner's knowledge, skills, and/or behaviour."

(ACHME 2011)

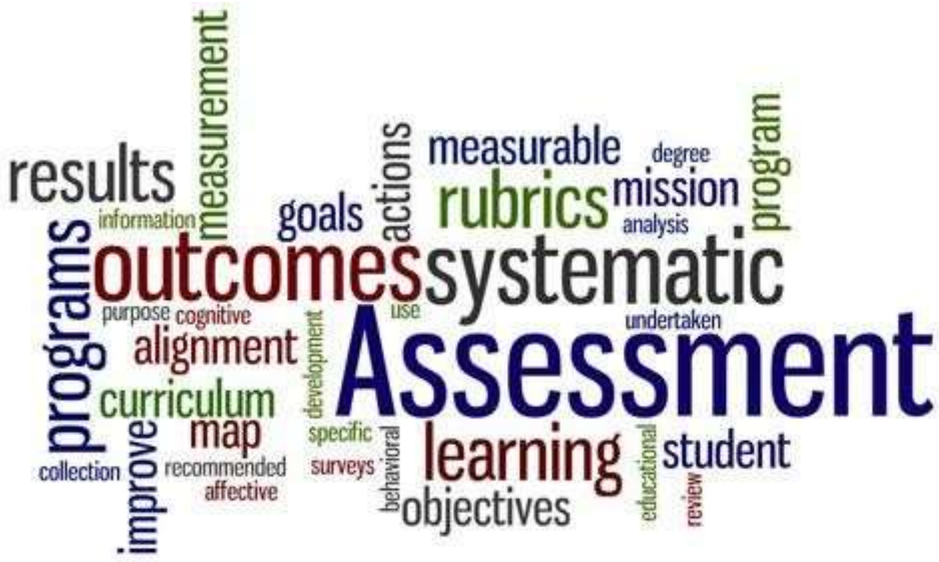

Main Purposes of Assessment (1):

- Determine the level of achievement of intended learning outcomes
- Promote Learning through motivation
- Provide feedback to student on their progress (formative and summative)
- A need to determine the competent from the non-competent candidates
- Educational tool to determine the cut-off point on the scoring scale which separates these categories
- Evaluate & provide feedback on effectiveness of teaching (QA)
- Certification- to ensure safe & competent professionals
- Licensure/Credentialing

© 2017 Knowledge E. All rights reserved. This workbook is intended for non-commercial use and may not be reproduced, distributed, or transmitted without the prior written permission of the publisher.

Effective Assessment:

• Alignment with learning outcomes (blueprint)

• Explicit and transparent assessment rubrics/policy

• Equitable for all students

• Multiple assessment methods

• Adequate sampling

• Validated methods of standard setting

• Timely feedback, balance between formative & summative

• Identification of underperforming students

• Regular review and quality assurance

• Consistency across teaching sites

Types of Assessment:

Formative

• concerned with student's ongoing educational progression, provide reassurance, promoting reflection & feedback, stimulate learning

• helping learners and assessors to know where they are and what they need to do to improve

• in the form of descriptive-feedback, must be timely, specific.

Summative

• judgemental, ensure standards, decisions are made on satisfactory completion, readiness to progress to next stage

• more appropriate to make high stakes decisions

© 2017 Knowledge E. All rights reserved. This workbook is intended for non-commercial use and may not be reproduced, distributed, or transmitted without the prior written permission of the publisher.

Essential criteria for a good tool of Assessment:

• Validity: Do they measure what was intended?

• Reliability: How consistent are the ratings?

• Feasibility/cost: Is the tool usable?

• Acceptability: Will people agree to use it?

• Educational impact: Does it have +ve influence?

Validity: (2)

• whether an instrument actually measures what it is supposed to measure

• the person developing the assessment instrument has to be sure that all items of the instrument are appropriate for the purpose of assessment

Reliability:

• refers to the extent to which assessments are consistent

• Assessments are usually expected to produce comparable outcomes, with consistent standards over time and between different learners and examiners.

Exercise: Match the answers on the targets

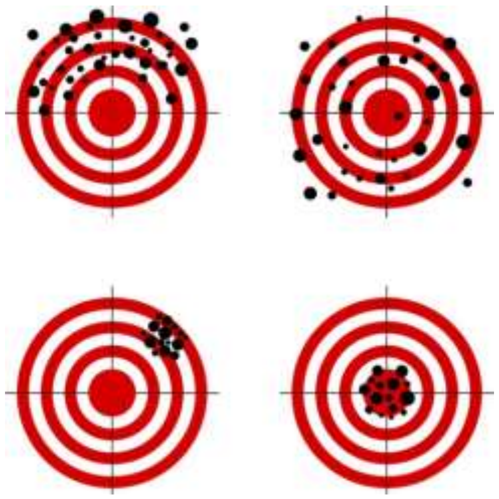

1. Unreliable & Unvalid
2. Reliable, Not Valid
3. Unreliable, but Valid
4. Both Reliable & Valid

© 2017 Knowledge E. All rights reserved. This workbook is intended for non-commercial use and may not be reproduced, distributed, or transmitted without the prior written permission of the publisher.

Aims of assessment:

- Validity: a multitude of methods needed
- Reliability: a lot of (combined) information is needed
- Learning impact: assessment should provide (longitudinal) information for learning

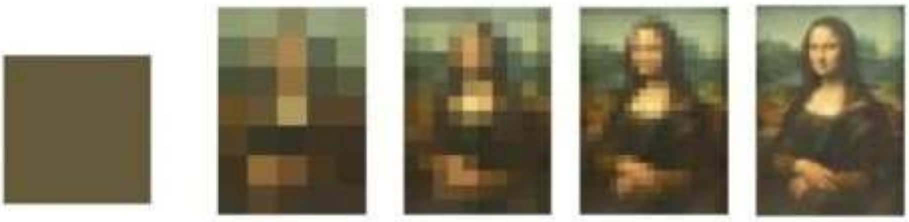

Assessment information as pixels → ultimately forming the whole picture

© 2017 Knowledge E. All rights reserved. This workbook is intended for non-commercial use and may not be reproduced, distributed, or transmitted without the prior written permission of the publisher.

Effective assessment methods

Type of Assessments:

- Multiple Choice (MCQ)
- Short answer questions (SAQ)
- Extended matching questions (EMQ)
- Script Concordance Test (SCT)
- Objective Structured Clinical Exam (OSCE)
- Mini Clinical Evaluation Exercise (Mini-CEX)
- Portfolio/Log book
- Direct Observation of Procedural Skills (DOPS)
- 360-degree assessment
- ......

The Miller's Pyramid (3)

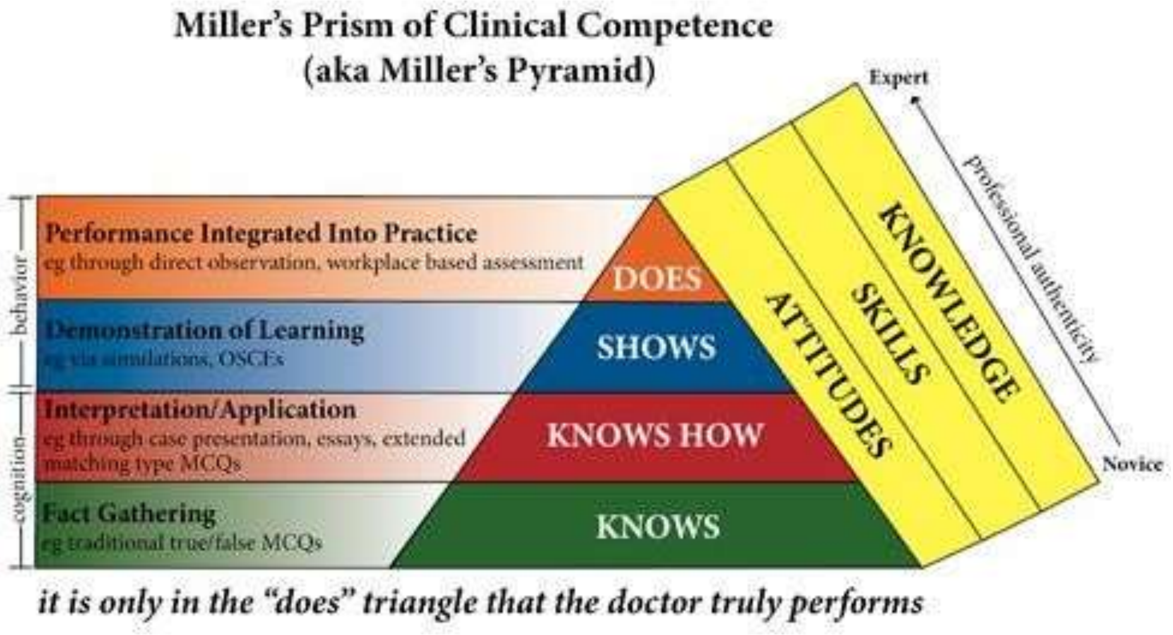

© 2017 Knowledge E. All rights reserved. This workbook is intended for non-commercial use and may not be reproduced, distributed, or transmitted without the prior written permission of the publisher.

MCQ

• Objectively test a wide range of student's knowledge

• If constructed properly, can assess higher order of thinking

• Easy to administer and mark using computer based testing & software

• Single best option (Type A) is the current trend used by the AMC, GMC, Specialist colleges

SAQ

• Clinical scenario followed by a few questions

• Student expect to respond in 1-3 words

• Can test core knowledge and some clinical reasoning

• Unlike MCQ, does not simply test recognition of the correct answer

• should be scenario based.

• A variety of styles may be used. e.g. brief answer, differential diagnoses, investigations, management options, pharmacological treatment options.....

• 0.5 mark for 1 key word, 1 mark for short answers, 2-7 marks per SAQ

Sample:

Name the spinal cord segments tested by asking the patient to perform the following maneuvers.

(1 mark each, max 3 marks)

A. Standing from a squat - L3-L4

B. Standing on their heels - L4-L5

C. Standing on their toes - S1

EMQ (1)

• Similar to MCQ except that the respond list consists of 15-20 options related to the topic in question

• Advantage: the effect of cuing as found in MCQ is minimised

• Easy for computer marking

© 2017 Knowledge E. All rights reserved. This workbook is intended for non-commercial use and may not be reproduced, distributed, or transmitted without the prior written permission of the publisher.

Example 1:

Theme: Back pain

Option list:

A. Ankylosing spondylitis.
B. Aortic dissection.
C. Prolapsed intervertebral disc.
D. Lumbar spondylosis.
E. Vertebral fracture.
F. Intervertebral disc infection.
G. Pars interarticularis defect.
H. Metastatic malignancy.

Lead-in statement:

For each patient with back pain, select the most likely diagnosis.

Stems:

Question 1

A 23-year-old man has a 6-month history of lower back pain. His pain is predominantly at the thoracolumbar junction and in the right buttock. The pain is worse in the morning and he has difficulty in getting out of bed. There is some improvement during the day. Examination shows restriction of lumbar spinal movements, particularly lateral flexion.

(Answer: A)

Question 2

A 32-year-old lady presents with acute onset of low back pain. The pain is constant and is not significantly affected by posture. All spinal movements are painful and difficult. Three weeks earlier, she had a urinary tract infection, which had been treated with amoxicillin.

(Answer: F)

Script Concordance Test (SCT)

- Relatively new format for assessing clinical reasoning
- Brief clinical scenario and students asked to make judgements regarding diagnostic possibilities or management options
- Certain amount of uncertainty to simulate real-life authentic clinical situations

© 2017 Knowledge E. All rights reserved. This workbook is intended for non-commercial use and may not be reproduced, distributed, or transmitted without the prior written permission of the publisher.

OSCE

• Introduced in 1975

• Now recognised as the gold standard for assessment of clinical competence

• Students rotate around a series of stations

• Standardised patients/actors in stations

• Perform tasks like: history taking, physical examination, procedures or communication / explanation to patients

• Subjective bias reduces with examiners and patients (simulated) standardised/calibrated

Method reliability as a function of testing time

Testing Time in Hours	MCQ[1]	Case-Based Short Essay[2]	PMP[1]	Oral Exam[3]	Long Case[4]	OSCE[5]	Mini CEX[6]	Practice Video Assess-ment[7]	In-cognito SPs[8]
1	0.62	0.68	0.36	0.50	0.60	0.54	0.73	0.62	0.61
2	0.76	0.73	0.53	0.69	0.75	0.69	0.84	0.76	0.76
4	0.93	0.84	0.69	0.82	0.86	0.82	0.92	0.93	0.92
8	0.93	0.82	0.82	0.90	0.90	0.90	0.96	0.93	0.93

[1]Norcini et al., 1985
[2]Stalenhoef-Halling et al., 1990
[3]Swanson, 1987
[4]Wass et al., 2001
[5]Van der Vleuten, 1988
[6]Norcini et al., 1999
[7]Ram et al., 1999
[8]Gorter, 2002

Clinical and performance based assessment

• Workplace Based Assessment (WPBA)

• Mini-CEX

• Portfolio / log books

• 360-degree assessment

• DOPS

© 2017 Knowledge E. All rights reserved. This workbook is intended for non-commercial use and may not be reproduced, distributed, or transmitted without the prior written permission of the publisher.

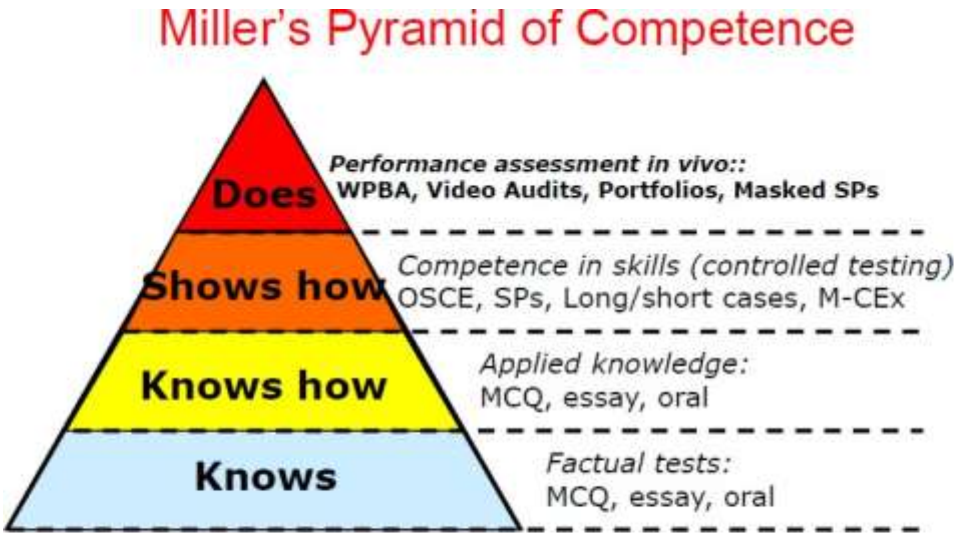

Miller GE. The assessment of clinical skills/competence/performance. Academic Medicine (Supplement) 1990; 65: S63-S7.

How to develop high quality MCQ questions

- Type A (Single Best Answer – SBA)

  Questions comprise a stem and 5 possible responses, only one of which is the best and most appropriately correct response.

  Optimally the span of optional responses should represent degrees of probability leading to one correct alternative in the circumstances outlined in the stem.

Other types of multiple choices

- Multiple scenario and multiple answer
- True and false
- Multiple true and false (Type J)
- Simple Combination answer
- Extended matched answer (EMQ)
- Multiple combination answer

© 2017 Knowledge E. All rights reserved. This workbook is intended for non-commercial use and may not be reproduced, distributed, or transmitted without the prior written permission of the publisher.

These other type of multiple choice questions are *less reliable* compared to Type A.

That is:

• good students perform less well and

• poor student better due to confusion inherent in the question style.

Type A MCQ: Is the preferred multiple-choice question style

Format of MCQs

• can only have one correct response (type A).

• include 5 possible answers

(i.e. 4 distracters)

• Need to be phrased in the positive, i.e. which one of the following is the correct option.

• "All of the above" or "None of the above" option not accepted.

• Cannot include multiple combination responses e.g. A & B are correct or A, C & E are correct.

The stem will improve the Type A question

• Clear wording

• Correct grammar

• CAPITALISE key words in the stem

• If stem a question, the responses should then each be answers and each response be complete in itself

• Concise stem

• Common wording

• Positive answers

• Clinical scenarios for senior years

• Information is specific to the population being assessed

© 2017 Knowledge E. All rights reserved. This workbook is intended for non-commercial use and may not be reproduced, distributed, or transmitted without the prior written permission of the publisher.

Exercise:

How to improve the quality of a MCQ?

© 2017 Knowledge E. All rights reserved. This workbook is intended for non-commercial use and may not be reproduced, distributed, or transmitted without the prior written permission of the publisher.

The distractors will improve the Type A question

- Equal length
- Correct grammar
- If stem a question, the responses should then each be answers and each response be complete in itself
- Concise distractors
- Avoid duplicating words

© 2017 Knowledge E. All rights reserved. This workbook is intended for non-commercial use and may not be reproduced, distributed, or transmitted without the prior written permission of the publisher.

Exercise:

How to improve the quality of a MCQ?

© 2017 Knowledge E. All rights reserved. This workbook is intended for non-commercial use and may not be reproduced, distributed, or transmitted without the prior written permission of the publisher.

In summary: A good multiple-choice question is...

• Type A

• Has a clear, concise, grammatically correct clinical stem

• Has distractors that are equal length, clear, avoid duplication and are grammatically correct.

Format of MCQs

Stem:

• Clinical scenario

• Which one of the following:
  - is the most likely diagnosis?
  - is the most likely aetiology?
  - is the most appropriate investigation?
  - is the most appropriate next step investigation?
  - is the most appropriate management?
  - is the most appropriate/best treatment?

What is the "cover test"?

• Cover all the options

• A good candidate should already have the correct answer in mind without looking at the options

Common test strategies used by 'test-wise' students:

• Rule of thumb: "Pick the longest answer."
  - Way to defeat this strategy: write answers of similar length, make the longest answer the wrong one

• Rule of thumb: "Pick the 'b' alternative."
  - Way to defeat this strategy: make sure each answer is used the same number of times, in random order.

• Rule of thumb: "Never pick an answer which uses the word 'always' or 'never' in it."
  - Way to defeat this strategy: do not use always or never in the alternatives.

© 2017 Knowledge E. All rights reserved. This workbook is intended for non-commercial use and may not be reproduced, distributed, or transmitted without the prior written permission of the publisher.

• Rule of thumb: "If there are two answers which express opposites, pick one or the other and ignore other alternatives."

- Way to defeat this strategy: sometimes offer opposites when neither is correct.

• Rule of thumb: "Pick a word which you remember was related to the topic."

- Way to defeat this strategy: when drawing up distracters (wrong answers) use terminology from the same area of the text as the right answer, but in distracters use those words incorrectly so the wrong answers are definitely wrong.

Developing Effective MCQ:

• Avoid longest option as correct answer

• Avoid absolutes e.g. “always”, “never”

• Avoid imprecise wordings e.g. “may”, “might”, “could”

• Avoid repeated wordings from stem

© 2017 Knowledge E. All rights reserved. This workbook is intended for non-commercial use and may not be reproduced, distributed, or transmitted without the prior written permission of the publisher.

Interactive session: writing high quality assessment items

Question 1:

Stem:

Which one of the following is the most likely?

Options:

A.

B.

C.

D.

E.

Answer:

Critique:

© 2017 Knowledge E. All rights reserved. This workbook is intended for non-commercial use and may not be reproduced, distributed, or transmitted without the prior written permission of the publisher.

Question 1:

Stem:

Which one of the following is the most likely?

Options:

A.

B.

C.

D.

E.

Answer:

Critique:

© 2017 Knowledge E. All rights reserved. This workbook is intended for non-commercial use and may not be reproduced, distributed, or transmitted without the prior written permission of the publisher.

Assessment blueprinting – aligning & integrating with the curriculum

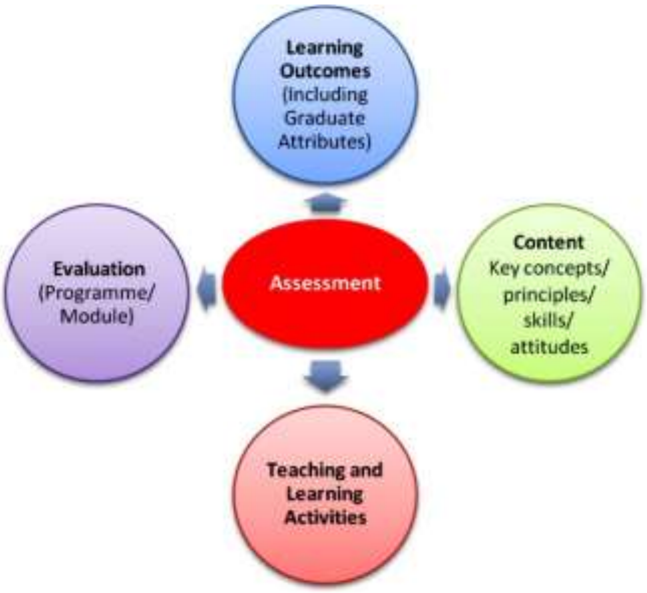

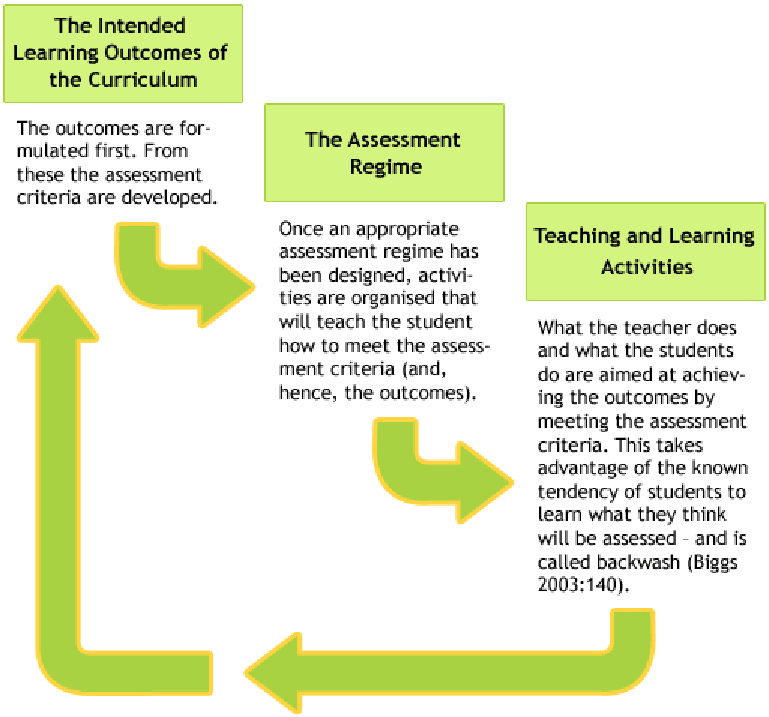

© 2017 Knowledge E. All rights reserved. This workbook is intended for non-commercial use and may not be reproduced, distributed, or transmitted without the prior written permission of the publisher.

Blueprint

Definition:

• is the matrix or chart reporting the number and type of test questions represented across the topics in content area, consistent with learning objectives and relative weight on test given to each topic.

Purpose of Blueprinting:

• to provide a conceptual map of examination format and the content area represented in assessment.

• Provides list of information about:

- Type of measurement tools and proportion of each question format in assessment shown in respective weighting column.
- Topics and the level of training for each topic and the relevant learning objectives.

• provides a readily available opportunity to vetting members to ensure that the essential topics are not overlooked

• provides an overview to assessment and can be readily used on stakeholders (particularly the external

• examiners, accreditation body) demand of quality assessment evident from its blueprint.

Sample Curriculum-Assessment Mapping

© 2017 Knowledge E. All rights reserved. This workbook is intended for non-commercial use and may not be reproduced, distributed, or transmitted without the prior written permission of the publisher.

Evaluation of items performance

Features of a good question

- Tests an important concept, skill or competency
- Discriminates between good and poor students
  - Higher performing students tend to get the question correct
  - Poorer students tend to get the question incorrect
- To achieve the above, the question should:
  - Relate to the curriculum
  - Be clear and not ambiguous (concise as possible, brief options)
  - The answer is clearly correct
  - Not too easy or too difficult
  - The other distractors offer a plausible alternative

Item performance statistics

P value

- In assessment, % students who got the correct response
- Measure of difficulty
- Very low or high P-values affect discriminatory ability (ceiling and floor effects) but acceptable if the question is testing a core 'must know' concept

Point Bi-Serial coefficient

- Measure of discriminatory ability
- measure of how well an item is able to distinguish between examinees who are knowledgeable and those who are not
- For an item that is highly discriminating, in general the examinees who responded to the item correctly also did well on the test
- Ranges from -1 to +1
- Correlation between the total mark for the exam (e.g. for the entire MCQ) and the student's response to the question which is marked as 0 (incorrect) or 1 (correct)

© 2017 Knowledge E. All rights reserved. This workbook is intended for non-commercial use and may not be reproduced, distributed, or transmitted without the prior written permission of the publisher.

- Desirable to have >0.2; >0.5 is difficult to achieve and is a hallmark of an excellent question
- Negative values indicate problem with question construction (e.g. confused the better performing students)

Examples of good questions

In patients taking Angiotensin-Converting Enzyme inhibitors, special care is required with the simultaneous prescription of:

A. thiazides

B. loop diuretics

C. potassium-sparing diuretics*

D. Mannitol

E. Digoxin

2011 MED1000: Point Bi-Serial 0.3

14/41/25/6/13

2012 MED1000: Point Bi-Serial 0.4

11/38/37/1/13

2014 MED1000: Point Bi-Serial 0.4

3/21/55/8/14

Which one of the following is a side-effect of atropine?

A. Constriction of the pupil

B. Decrease in heart rate

C. Increased gastrointestinal motility

D. Dry mouth*

E. Bronchoconstriction

2013 MED1000: Point Bi-Serial 0.3

18/13/10/52/7

© 2017 Knowledge E. All rights reserved. This workbook is intended for non-commercial use and may not be reproduced, distributed, or transmitted without the prior written permission of the publisher.

Sound waves are transmitted directly into the inner ear by movements of the:

A. Cochlea

B. Round window

C. Tympanic membrane

D. Auditory ossicles

E. Oval window*

2012 MED1000: Point Bi-Serial 0.3

0/10/9/35/46

© 2017 Knowledge E. All rights reserved. This workbook is intended for non-commercial use and may not be reproduced, distributed, or transmitted without the prior written permission of the publisher.

Standardising Assessment: formative and summative assessments

Standard Setting

• Any standard – absolute or relative – is based on some type of judgement.

• How good is good enough? Can only be answered by someone's judgement.

It is important that the judgements are;

- Made by persons who are qualified to make them
- Meaningful to the persons who are making them &
- Made in a way that takes into account the purposes of the test.

Livingstone & Zieky (1982)

Why set standards in assessment?

• Setting standards in assessment a relatively new area of study

• Reasons for setting standards

• Licensure

• Credentialing

• A need to determine the competent from the non-competent candidate

• Educational tool to determine the cut-off point on the scoring scale which separates these categories

Norm-referenced versus Criterion-referenced standards

Norm referenced

• Standard based upon performance of an external large representative sample (the norm group)

• Norm referenced approach employs a group referenced to a standard and will result in reasonable standards providing the group is large and heterogeneous.

• Set as the mean performance of the candidates OR by defining units of SD from mean.

• Students are compared with each other within the cohort

• Standards vary from year to year due to shifts in group ability.

• Always some candidates will FAIL.

© 2017 Knowledge E. All rights reserved. This workbook is intended for non-commercial use and may not be reproduced, distributed, or transmitted without the prior written permission of the publisher.

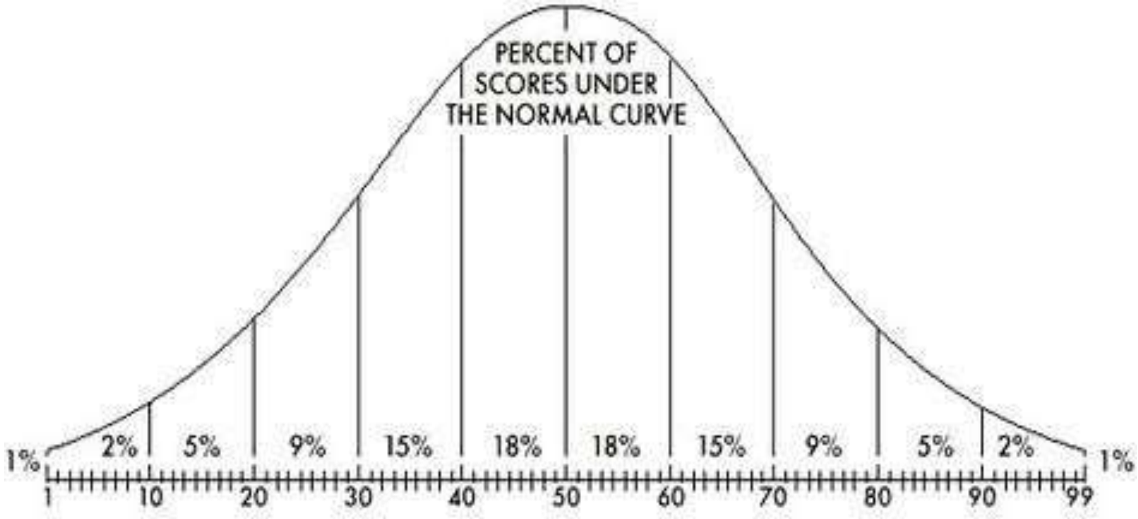

Criterion-referenced approach

- This links the standard to the content of the competence level under consideration
- A standard is an absolute level of knowledge and skill a student must possess in order to pass the stage/course
- Absolute criterion
- Stays the same over multiple administrations
- Will differ in pass marks year to year depending on the difficulty of the assessment

Criterion-referenced: Compensatory vs. Conjunctive Standards

- **Compensatory**
  - Standard set on total test score
  - Candidate can compensate for poor performance in one area with good performance in others
- **Conjunctive**
  - Standards set for individual components of examination
  - Candidates cannot compensate for poor performance in one area
  - Reliability of test components can be a problem (eg: OSCE single station versus entire exam)
  - Useful for feedback to candidates
  - Each test/skill component can be fed back separately to candidates
  - Good for formative assessment

© 2017 Knowledge E. All rights reserved. This workbook is intended for non-commercial use and may not be reproduced, distributed, or transmitted without the prior written permission of the publisher.

Standard Setting Methods

- **Angoff model**

Expert judges make estimates of borderline candidate

- **Ebel's model**

Judges categorise items in test to levels of difficulty and relevance. After classification, judges decide proportion of items in each category that a hypothetical group of candidates would respond correctly

- **Nidelsky model**

For MCQ

For each item, judges decide on how many distracters a minimally competent examinee would recognise as incorrect

- **Borderline group**

Judges identify actual borderline group. Median score of this group used as passing score

Selecting panellists

- **Panellists should be**
  - Experts in field of examination
  - Familiar with examination methods
  - Familiar with the curriculum/course
  - Familiar with level of candidates
  - Interested in education (teachers)
  - Best to be involved in the teaching

Modified Angoff

- Judgemental approach
- Judges assess performance of the borderline candidate
- Group of 'expert' judges make estimates on how the borderline candidate would perform on the test items.
- Estimates are discussed
- Highest and lowest score justify their stance and consensus reached.
- The consensus mark is set as the borderline / pass mark.
- Commonly used for MCQ, SAQ, OSCE exams

© 2017 Knowledge E. All rights reserved. This workbook is intended for non-commercial use and may not be reproduced, distributed, or transmitted without the prior written permission of the publisher.

Calibration Exercise

Modified Angoff

Question 1

A midline neck swelling that moves with tongue protrusion is most likely to be which one of the following?

A. Carotid artery aneurysm

B. Dentigerous cyst

C. Lipoma

D. Sebaceous cyst

E. Thyroglossal duct cyst

Your Borderline:

Question 2

A young man is holding a cup of coffee in his hand as he converses with a friend.

As fatigue develops in his biceps muscle which of the following changes in neural activation would you expect?

A. Increased activation of the deltoid muscle

B. Decreased concentric activation of triceps muscle

C. Increased concentric activation of the triceps muscle

D. Increased inhibition of the biceps motor neurone pool

E. Increased excitation of the triceps motor neurone pool

Your Borderline:

© 2017 Knowledge E. All rights reserved. This workbook is intended for non-commercial use and may not be reproduced, distributed, or transmitted without the prior written permission of the publisher.

Question 3

Which bone is arrowed on the illustration?

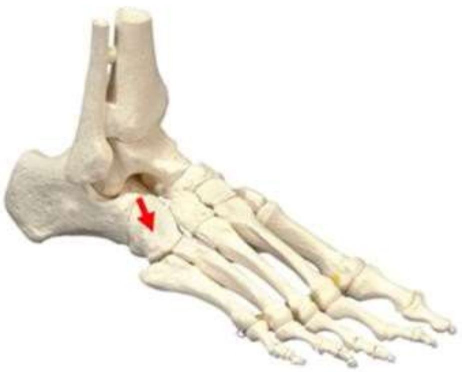

A. The calcaneus
B. The cuboid
C. The lateral cuneiform
D. The navicular
E. The talus

Your Borderline:

Nidelsky model (MCQ)

- Used for MCQ only
- For each item, judges decide on how many distracters a minimally competent examinee would recognise as incorrect
- Reach consensus
- Borderline mark 0.2 – 1.0
- Sum of borderline marks = pass mark for the MCQ paper

Probability

- 1/5 0.2
- ¼ 0.25
- 1/3 0.33
- ½ 0.5
- 1/1 1.0

© 2017 Knowledge E. All rights reserved. This workbook is intended for non-commercial use and may not be reproduced, distributed, or transmitted without the prior written permission of the publisher.

Exercise:

A 30-year-old woman presents to the GP clinic. She is 12 weeks pregnant by certain dates and that 2 hours earlier she passed a moderate amount of blood with clots per vagina, which was associated with crampy lower abdominal pain. On vaginal examination the cervical canal admits one finger readily and bimanual examination reveals a uterus compatible in size with a pregnancy of only eight weeks duration.

What is the MOST LIKELY diagnosis?

A Threatened abortion

B Cervical incompetence

C Incomplete abortion (correct response)

D Ectopic pregnancy

E Missed abortion

What is the Borderline mark? __________

© 2017 Knowledge E. All rights reserved. This workbook is intended for non-commercial use and may not be reproduced, distributed, or transmitted without the prior written permission of the publisher.

Interactive session: Standard setting the borderline/pass mark

Question 1:

A 65-year-old man presents to the surgical pre-admission clinic for assessment prior to repair of an uncomplicated inguinal hernia.

His assessment reveals a history of cardiovascular disease. In assessing his peri-operative risk factors, which one of the following would be most concerning regarding an increased risk of peri-operative complications?

A. Stable angina pectoris

B. Shortness of breath on exertion

C. Occasional premature ventricular contractions on ECG

D. Acute myocardial infarction 2 years ago

E. Taking metoprolol 100mg twice daily

Your Borderline:

Nidelsky: ________________

Modified Angoff: __________________

Question 2:

A 3-year-old girl has suspected bacterial meningitis. Which one of the following CSF findings would be most consistent with this diagnosis?

A. clear looking CSF

B. negative PCR (Polymerase Chain Reaction) analysis

C. 32 x 106 /L neutrophils

D. increased lymphocyte count

E. reduced CSF:blood glucose ratio

Your Borderline:

Nidelsky: ________________

Modified Angoff: __________________

© 2017 Knowledge E. All rights reserved. This workbook is intended for non-commercial use and may not be reproduced, distributed, or transmitted without the prior written permission of the publisher.

Question 3:

A 30-year-old woman presents with jaundice and malaise.

Serology results are as follow:

HAV-IgM	negative
HBsAg	positive
anti-HBc	positive
HBV-DNA	positive
anti-HCV	negative

Which one of the following is the most likely underlying diagnosis?

A. Acute hepatitis A

B. Acute hepatitis B

C. Acute hepatitis C

D. Chronic hepatitis B

E. Chronic hepatitis C

Your Borderline:

Nidelsky: ________________

Modified Angoff: ___________________

Question 4:

A 26-year-old man is admitted to the Emergency Department after a motor vehicle accident. He has sustained several fractured ribs anteriorly. His voice is hoarse. Chest X-ray shows a widened mediastinum and obliteration of the aortic knob. ECG is normal.

Which one of the following is the most appropriate next step in management?

A. Echocardiogram (19%)

B. Laryngoscopy (1%)

C. Intubation and mechanical ventilation (12%)

D. Chest CT with contrast (38%)

E. Pericardiocentesis (30%)

Your Borderline:

© 2017 Knowledge E. All rights reserved. This workbook is intended for non-commercial use and may not be reproduced, distributed, or transmitted without the prior written permission of the publisher.

Nidelsky: ________________

Modified Angoff: __________________

Question 5:

A 40-year-old woman presented with complaints of fever, weight loss and shortness of breath. During investigation the lung was biopsied and the pathologist made a diagnosis of sarcoidosis.

What pathological changes are most likely to be seen in the biopsy?

A. Chronic interstitial inflammation with eosinophilic infiltration (19%)

B. Multiple small and medium-size vessel walls with necrosis and giant cells (1%)

C. Non caseating granulomata along the lymphatics around vessels and bronchi (48%)

D. Large numbers of pigmented macrophages within respiratory bronchioles (1%)

E. Chronic interstitial inflammation with multiple loosely-formed interstitial granulomas (31%)

Your Borderline:

Nidelsky: _______________

Modified Angoff: _________________

Setting the pass mark for OSCE

• OSCE: borderline regression (best method)

• Borderline regression is a method of standard setting

• For each station, the scores of each candidate plot on a graph, grouping them by the overall global judgement each candidate received

• then draw a regression line through each group of scores and where the line intersects with the borderline group - the passing score for the station

© 2017 Knowledge E. All rights reserved. This workbook is intended for non-commercial use and may not be reproduced, distributed, or transmitted without the prior written permission of the publisher.

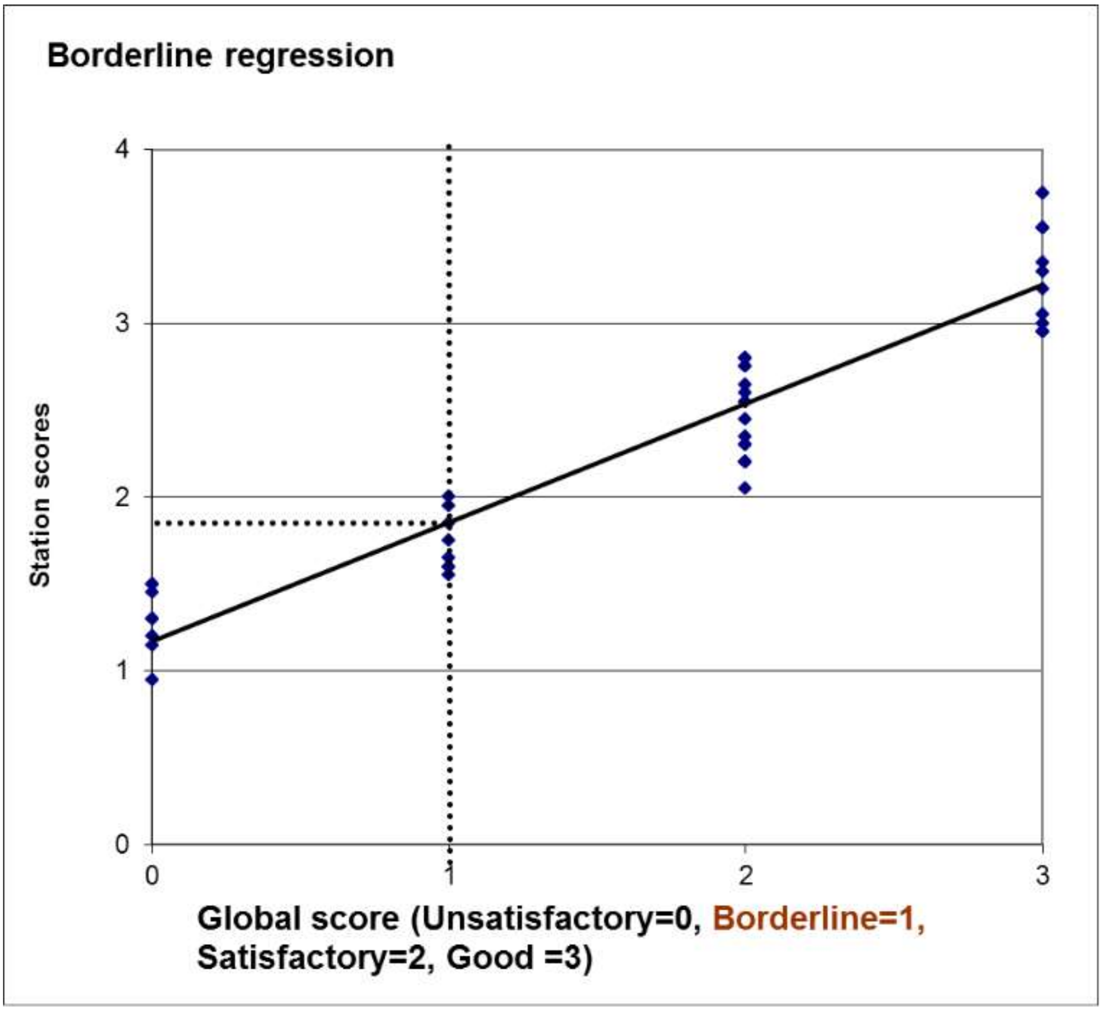

http://www.gmc-uk.org/PLAB___Borderline_regression_pilot___guidance_for_examiners___DC7576.pdf_61114462.pdf

Educational benefits standard setting

- Faculty development

Allows faculty to compare their expectation of candidate performance to actual performance

- Quality of test materials

Ensures scrutiny of test materials 'quality control process'

- Opportunity for multi-disciplinary review and discussion of examination questions (validity)
- Quality control process of examination materials.
- Accountability
- Defensibility
- Fairness / equity

© 2017 Knowledge E. All rights reserved. This workbook is intended for non-commercial use and may not be reproduced, distributed, or transmitted without the prior written permission of the publisher.

Workshop: Using SCT to assess and enhance clinical reasoning

Overview:

1. The use of Script Concordance Test (SCT) to assess Clinical Reasoning (CR)
2. How to develop high quality SCT items in different health disciplines
3. Practice session to develop some new SCTs
4. Reference panel scoring and discussions
5. Using SCT in the formative assessment to enhance learning

The use of SCT to assess Clinical Reasoning (CR)

What is Clinical reasoning (CR)?

• is defined as "the sum of the thinking and decision-making processes associated with clinical practice"

• the process by which the clinician collect cues, process the information, come to an understanding of a patient problem or situation, plan and implement interventions, evaluate outcomes, and reflect on and learn from the process

• A sound knowledge base for basic clinical science and an understanding of disease pathophysiology are required for the effective learning of CR

• CR is a foundation requirement for entry level clinical practice education and cannot be assumed to develop in the absence of fundamental educational strategies

• effective CR skills have a positive impact on patient outcomes

Hoffman, 2007; Kraischsk & Anthony, 2001; Laurie et al., 2001

Cognitive capabilities

• hypothetico-deductive reasoning

• pattern recognition

Hypothetico-deductive reasoning

• data collection, hypotheses generation, data interpretation followed by hypothesis evaluation

• more suitable for the novice learners (e.g. medical students and interns)

© 2017 Knowledge E. All rights reserved. This workbook is intended for non-commercial use and may not be reproduced, distributed, or transmitted without the prior written permission of the publisher.

Pattern Recognition

• clinicians identifying patterns and clinical presentations of a disease and then comparing them with what they have stored in their long term memory to come up with the diagnosis

• this model requires significantly more clinical experiences in order to be accurate

• therefore more commonly used in the expert specialists

Assessment of CR (5)

• Script Concordance Testing (SCT) is a relatively new modality in assessing clinical reasoning (CR)

• candidates are presented with a clinical scenario, followed by a new piece of information (e.g. history, examination findings, and investigations)

• then asked to assess whether this additional information increases or decreases the probability of the suggested provisional diagnosis, or increases or decreases the usefulness of a proposed investigation or management option

A. **CLINICAL SCENARIO:**

James aged 25 years, presents with chest pain and shortness of breath. He came back from USA yesterday. On examination, his BP is 110/90, pulse 120/min.

	**If you were thinking of the diagnosis**	**And then you find**	**The diagnosis is**	
1.	Community acquired pneumonia	A normal chest radiograph	A B C D E<br>-2 -1 0 +1 +2<br>Weighted Score<br>A: 1.0, B: 1.0, C: 0.3	
2.	Pneumothorax	Marfan syndrome	A B C D E<br>-2 -1 0 +1 +2<br>Weighted Score<br>C: 0.1, D: 1.0, E: 0.3	-2 = effectively rules out<br>-1 = less likely<br>0 = neither less nor more likely<br>+1 = more likely<br>+2 = the working diagnosis
3.	Pulmonary embolism	Swollen left leg with dilated veins	A B C D E<br>-2 -1 0 +1 +2<br>Weighted Score<br>D: 0.2, E: 1.0	

© 2017 Knowledge E. All rights reserved. This workbook is intended for non-commercial use and may not be reproduced, distributed, or transmitted without the prior written permission of the publisher.

Scenario C

A 35-year-old woman comes to you complaining of a painful area in her left breast, of 4 days duration. She had a normal menses finishing 3 days ago; she does not take any medication. Her grandmother had breast cancer at age 65, no other relative has a history of breast cancer but her mother has had a number of cysts removed from both breasts over the years.

<table>
<tr><th></th><th>If you were considering the following treatment or action</th><th>And then you find:</th><th>You would then consider the treatment or action to be</th><td rowspan="4">-2 = much less appropriate

-1 = slightly less appropriate

0 = neither less nor more appropriate

+1 = slightly more appropriate

+2 = much more appropriate</td></tr>
<tr><td>57.</td><td>Waiting and reviewing in 3 months</td><td>There is a firm, mobile mass in the axillary tail of the left breast</td><td>A B C D E
-2 -1 0 +1 +2</td></tr>
<tr><td>58.</td><td>Ordering a mammogram</td><td>There is a firm, indurated, tender mass in the left upper outer quadrant and that she is breastfeeding her 6 month old infant</td><td>A B C D E
-2 -1 0 +1 +2</td></tr>
<tr><td>59.</td><td>Ordering a fine needle biopsy of the painful area of the breast</td><td>There is no palpable abnormality</td><td>A B C D E
-2 -1 0 +1 +2</td></tr>
</table>

© 2017 Knowledge E. All rights reserved. This workbook is intended for non-commercial use and may not be reproduced, distributed, or transmitted without the prior written permission of the publisher.

- the process reflects the clinician's everyday real-world decision-making processes
- tests CR and problem-solving ability, and how clinicians apply their learned knowledge in the context of uncertainty
- to score these questions, the candidate's decision is compared to that of a reference panel of experts in the field
- the test has been shown to be valid and reliable (6-9)

Panel Scoring

**Score Key**	**-2**	**-1**	**0**	**+1**	**+2**
Number of panelist choosing the answer (out of 10)	7	2	1	0	0
Formula	7/7	2/7	1/7	0/7	0/7
Candidate score	1	0.29	0.14	0	0

A. **CLINICAL SCENARIO:**

James aged 25 years, presents with chest pain and shortness of breath. He came back from USA yesterday. On examination, his BP is 110/90, pulse 120/min.

	**If you were thinking of the diagnosis**	**And then you find**	**The diagnosis is**	
**1.**	Community acquired pneumonia	A normal chest radiograph	**A B C D E**<br>-2 -1 0 +1 +2<br>Weighted Score<br>A: 1.0, B: 1.0, C: 0.3	-2 = effectively rules out
**2.**	Pneumothorax	Marfan syndrome	**A B C D E**<br>-2 -1 0 +1 +2<br>Weighted Score<br>C: 0.1, D: 1.0, E: 0.3	-1 = less likely<br>0 = neither less nor more likely<br>+1 = more likely
**3.**	Pulmonary embolism	Swollen left leg with dilated veins	**A B C D E**<br>-2 -1 0 +1 +2<br>Weighted Score<br>D: 0.2, E: 1.0	+2 = the working diagnosis

© 2017 Knowledge E. All rights reserved. This workbook is intended for non-commercial use and may not be reproduced, distributed, or transmitted without the prior written permission of the publisher.

Developing SCTs (10)

• In order to allow the students to choose from the full range of the 5 response options, "much less likely (-2)" rather than "ruling out the diagnosis"; and "much more likely (+2)" rather than "definitive diagnosis" are used in the questions

Interactive Session

Writing actual SCT questions

Template

**<u>Clinical Scenario A</u>**				
	If you were thinking of...	and then you find that…	this hypothesis becomes …	**-2** = much less likely<br>**-1** = less likely<br>**0** = neither less nor more likely<br>**+1** = more likely<br>**+2** = much more likely
**1.**			**A B C D E**<br>-2 - 1 0 +1 +2	
**2.**			**A B C D E**<br>-2 - 1 0 +1 +2	
**3.**			**A B C D E**<br>-2 - 1 0 +1 +2	
**4.**			**A B C D E**<br>-2 - 1 0 +1 +2	

© 2017 Knowledge E. All rights reserved. This workbook is intended for non-commercial use and may not be reproduced, distributed, or transmitted without the prior written permission of the publisher.

Clinical Scenario B

	If you were thinking of...	and then you find that…	this hypothesis becomes …	
**1.**			**A B C D E**<br>-2 - 1 0 +1 +2	
**2.**			**A B C D E**<br>-2 - 1 0 +1 +2	**-2** = much less likely<br>**-1** = less likely<br>**0** = neither less nor more likely<br>**+1** = more likely<br>**+2** = much more likely
**3.**			**A B C D E**<br>-2 - 1 0 +1 +2	
**4.**			**A B C D E**<br>-2 - 1 0 +1 +2	

© 2017 Knowledge E. All rights reserved. This workbook is intended for non-commercial use and may not be reproduced, distributed, or transmitted without the prior written permission of the publisher.

Selecting the Reference Panel (5)

• a panel of 10-15 expert members relevant to the discipline is recommended to produce credible and reliable scores

• composition should include clinical teachers and academics who are familiar with the curriculum and experts in the field relevant to the discipline

• panel scores could be collected online to avoid getting all members in one meeting

How to choose SCT items according to panelist responses?

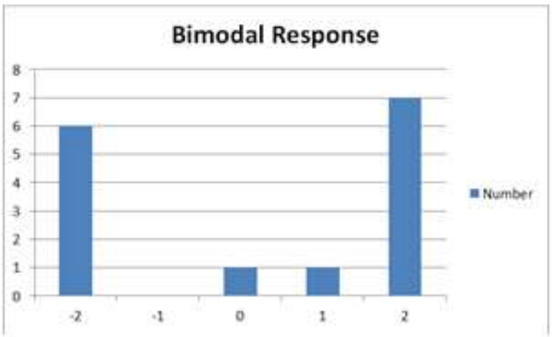

Fig 2.

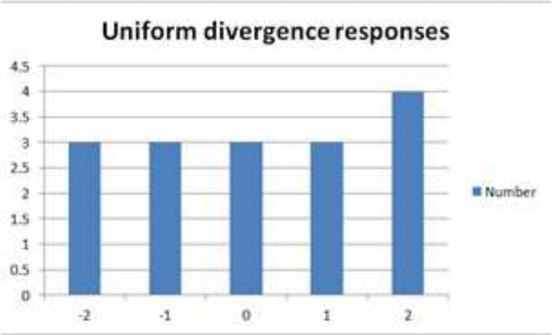

Fig 3.

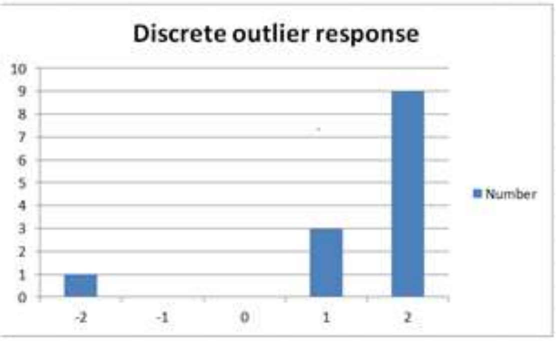

Fig 4.

Wan SH. Review: Using Script Concordance Test to Assess Clinical Reasoning Skills in Undergraduate and Postgraduate Medicine. Hong Kong Med J. 2015 Oct;21(5):455-61. doi: 10.12809/hkmj154572. Epub 2015 Aug 28.

© 2017 Knowledge E. All rights reserved. This workbook is intended for non-commercial use and may not be reproduced, distributed, or transmitted without the prior written permission of the publisher.

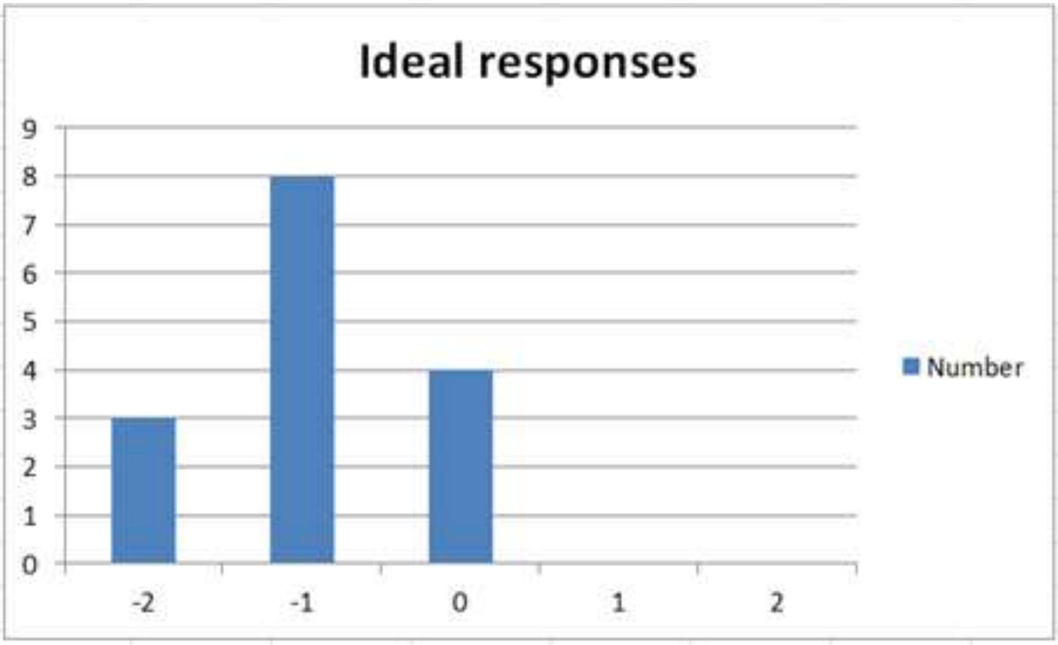

Ideal SCT responses

Practice session

Scoring some SCTs

Scenario: A

Scenario: B

Scenario: C

© 2017 Knowledge E. All rights reserved. This workbook is intended for non-commercial use and may not be reproduced, distributed, or transmitted without the prior written permission of the publisher.

Setting the pass mark:

• the expert panels' mean scores and standard deviations (SD) are chosen to guide the process

• calculated by asking all the members of the panel to attempt the same set of SCT questions

• borderline pass score of the undergraduate students are usually set at 3 to 4 SDs below the expert panel's mean score.

Using SCT in formative assessment to enhance learning

Formative assessment

• expert panel consensus scores are provided to the candidates;

• followed by expert clinicians explaining and discussing the options in each scenario with the candidates for constructive feedback

• enhance clinical reasoning learning

• SCT could identify borderline students with sub-optimal CR skills where appropriate remedial measures could be provided

Summative assessment

• particularly in context where there isn't a large pool of SCT items, it is important not to release or discuss panel's responses

• unlike MCQ where there is only one SBA, the partial credit scoring model in SCT makes sharing of 'correct' answers after the examination difficult

© 2017 Knowledge E. All rights reserved. This workbook is intended for non-commercial use and may not be reproduced, distributed, or transmitted without the prior written permission of the publisher.

Evaluation of student performance in assessment

Identification of underperforming students

• Early identification by reviewing formative and in-course continuous assessments

• Individual face to face discussion with year coordinator, domain or discipline head

• Involvement of Academic Support Team, Student Life

• Early remedial support and progress monitoring

Clinical and performance based assessment

Workplace Based Assessment – WPBA

• VALIDITY – what you do every day with the students

• Formalisation of the informal – contemporaneous feedback

• 1 : 1 feedback with students

• Very useful formative tool to inform learning and make suggestions for improvement

Mini-CEX

• Authentic workplace based patient encounter

• In patient, outpatient or ED settings

• Examiner watches the examinee taking a focused history, physical exam, provide diagnosis or management plan

• 15-20 mins followed by 5 min feedback

• Repeated a few times during each rotation

How to do a Mini-CEX

• Students' responsibility to get at least 2 Mini-CEXs per rotation. Recommend to have 1 Mini-CEX per week (4-5 per rotation)

• Choose only 1 task for each Mini-CEX (e.g. just history, just exam or just management discussion) to observe

• Rate the students' clinical competencies according to the guide in the form

• Give structured and constructive feedback after the observation

• More importantly - Work out a plan for recommended improvement!

© 2017 Knowledge E. All rights reserved. This workbook is intended for non-commercial use and may not be reproduced, distributed, or transmitted without the prior written permission of the publisher.

Portfolio/Log book

• Collection of evidence that learning has taken place

• Include reflections on the experiences and a description of the further learning that has resulted

• Include also: practical procedure log, written evaluations (e.g. Mini-CEX, MSF)

• Can assess competencies and professionalism

DOPS - Direct Observation of Procedural Skills

• Variation of Mini-CEX

• Feedback and access competencies in procedural skills

• Usually in the actual work place on 'real patients' e.g. CVP insertion, intubation, ABG sampling, etc.

• Commonly used in post-graduate education and fellowship training

© 2017 Knowledge E. All rights reserved. This workbook is intended for non-commercial use and may not be reproduced, distributed, or transmitted without the prior written permission of the publisher.

360-degree assessment (1)

- Also known as Multisource Feedback (MSF)
- Commonly used in post-graduate education and fellowship training
- Evidence systematically collected from a number of individuals in a legitimate position to make a judgement about the trainee's performance (senior colleagues, nurses, other health care workers, patients)
- Ratings remain anonymous
- Aim to provide fair and balanced view of trainee's abilities and professional behaviour

International Medical Graduate Assessment Form — page 1

Medical Colleague Questionnaire

Assessed Doctor's name:

How well would you describe your professional relationship to this doctor? (mark one)

☐ Not at all ☐ Not well ☐ Somewhat ☐ Well ☐ Very well

Please rate your colleague on the performance statements according to the following scale. Please use U/A if you have insufficient information to respond to an item. Your individual replies will remain confidential. Replies from all medical colleagues will be combined before feedback is given to the doctor.
(Please mark (✓) with an ink pen)

This doctor :

Statement	☐ 0 Unable to assess	☐ 1 I strongly disagree	☐ 2 I disagree	☐ 3 Neutral	☐ 4 I agree	☐ 5 I strongly agree
1. Communicates well with patients.	☐	☐	☐	☐	☐	☐
2. Reaches the correct diagnosis in a timely manner.	☐	☐	☐	☐	☐	☐
3. Refers patients appropriately.	☐	☐	☐	☐	☐	☐
4. Provides appropriate information for colleagues to provide follow-up patient care.	☐	☐	☐	☐	☐	☐
5. Accepts responsibility for care of ongoing issues.	☐	☐	☐	☐	☐	☐
6. Provides pertinent and timely information about patients when required.	☐	☐	☐	☐	☐	☐
7. Makes appropriate use of community resources for patient management (for example, public health, social services, mental health services)	☐	☐	☐	☐	☐	☐
8. Participates in a system to provide care for patients outside of regular office hours.	☐	☐	☐	☐	☐	☐
9. Recognises and takes action when urgent intervention is required.	☐	☐	☐	☐	☐	☐
10. Takes responsibility for actions and decisions.	☐	☐	☐	☐	☐	☐
11. Demonstrates appropriate clinical judgment.	☐	☐	☐	☐	☐	☐
12. Maintains patient confidentiality.	☐	☐	☐	☐	☐	☐
13. Works well with colleagues.	☐	☐	☐	☐	☐	☐
14. Speaks respectfully of colleagues in conversations with patients and co-workers.	☐	☐	☐	☐	☐	☐

15. Documents care appropriately.

© 2017 Knowledge E. All rights reserved. This workbook is intended for non-commercial use and may not be reproduced, distributed, or transmitted without the prior written permission of the publisher.

International Medical Graduate Assessment Form

page 2

Medical Colleague Questionnaire

This doctor:

16. Is willing to take responsibility for error.

☐ **0**	☐ **1**	☐ **2**	☐ **3**	☐ **4**	☐ **5**
Unable to assess	I strongly disagree	I disagree	Neutral	I agree	I strongly agree

17. Contributes to administrative practices supporting good medical care (office protocols, timely reports/information flow).

☐ **0**	☐ **1**	☐ **2**	☐ **3**	☐ **4**	☐ **5**
Unable to assess	I strongly disagree	I disagree	Neutral	I agree	I strongly agree

18. Writes prescription and orders clearly.

☐ **0**	☐ **1**	☐ **2**	☐ **3**	☐ **4**	☐ **5**
Unable to assess	I strongly disagree	I disagree	Neutral	I agree	I strongly agree

19. Demonstrates commitments to health promotion in the community and practice.

☐ **0**	☐ **1**	☐ **2**	☐ **3**	☐ **4**	☐ **5**
Unable to assess	I strongly disagree	I disagree	Neutral	I agree	I strongly agree

20. Manages health care resources appropriately.

☐ **0**	☐ **1**	☐ **2**	☐ **3**	☐ **4**	☐ **5**
Unable to assess	I strongly disagree	I disagree	Neutral	I agree	I strongly agree

21. Appears committed to and current with advances in medical education.

☐ **0**	☐ **1**	☐ **2**	☐ **3**	☐ **4**	☐ **5**
Unable to assess	I strongly disagree	I disagree	Neutral	I agree	I strongly agree

22. Appears medically capable to practise medicine. (is unencumbered by physical and mental health problems and drug, alcohol or substance abuse.)

☐ **0**	☐ **1**	☐ **2**	☐ **3**	☐ **4**	☐ **5**
Unable to assess	I strongly disagree	I disagree	Neutral	I agree	I strongly agree

23. Is someone I would recommend to a friend or family member.

☐ **0**	☐ **1**	☐ **2**	☐ **3**	☐ **4**	☐ **5**
Unable to assess	I strongly disagree	I disagree	Neutral	I agree	I strongly agree

An overall rating for this candidate's performance and professionalism in all areas.

**1**	**2**	**3**	**4**	**5**	**6**	**7**	**8**	**9**
	**Unsatisfactory**			**Satisfactory**			**Superior**	

This questionnaire was developed with funding from the Medical Council of Canada in a study led by Jocelyn Lockyer, University of Calgary, Alberta Canada.

© 2017 Knowledge E. All rights reserved. This workbook is intended for non-commercial use and may not be reproduced, distributed, or transmitted without the prior written permission of the publisher.

Giving feedback to students to ensure lasting knowledge

Feedback is:

- Specific information about the comparison between a learner's observed performance and a standard, given with the intent to improve the learner's performance
- "A conversation about performance"
- Guidance to develop lifelong learning

Why is feedback important?

- Acknowledge of learning
- Framework for development
- Provides motivation
- Use reflective practice to support giving feedback well
- Debrief following the incident
- Be supportive but honest, all doctors are accountable for their practice and how they learn from mistakes

Effective Feedback:

- Ask for self-reflection
- Be Positive
- Objective, specific, genuine
- Suggestion on areas of improvement
- Take home message
- Leads to reassessment
- Best way to enhance future learning

Tips:

- Recipient: what went well
- Provider: what also went well
- Recipient: opportunities for changes improvement
- Provider: opportunities for changes

© 2017 Knowledge E. All rights reserved. This workbook is intended for non-commercial use and may not be reproduced, distributed, or transmitted without the prior written permission of the publisher.

Are they ready?

- Formative opportunity in each Mini-CEX session
- Student select the case and discuss with you what task(s) to focus on
- You as the assessor: critique, comment and discuss

Hints & tips for giving feedback:

- Empathic, respectful and direct
- Ask the student to reflect:
- What do you think went well?
- What are you thinking when you ask/did...?
- Started off with positive comments:
    - I like your communication skills, eye contact with patient,....
- Acknowledge the difficulties encountered:
    - This is a difficult / complex case...
    - I know you have been trying your best to....
    - I understand your position..
    - I can see why you....
- Feedback sandwich:
    - Positive followed by negative comments or areas of improvement
- When giving negative feedback:
    - "Don't feel bad about this... helping you to improve...."
- Pointing out errors:
    - Opportunities for the others to learn from mistakes
- Suggestions for improvement:
    - "You could do better in the future if......"

© 2017 Knowledge E. All rights reserved. This workbook is intended for non-commercial use and may not be reproduced, distributed, or transmitted without the prior written permission of the publisher.

Action plan:

- 1-2 dot points about future action: e.g. "practice on CVS examination skills, review on management of acute GIB…"
- Let's try it next time…., We can move forward by…

Calgary Cambridge Model (12)

- Start with the learner's agenda
- Focus on outcome required
- Encourage self-assessment & problem solving
- Provide balanced feedback
- Be respectful
- Structure and summarise
- Action plan

Training video

https://vimeo.com/90176859

In-effective Feedback

- Negative criticism – e.g. you are hopeless, who taught you this! I have never seen….. "But…"
- No suggestions for ways of improvement
- Convoluted comments

Watch this video and critique about the assessor

https://vimeo.com/90176858

Your critique about this assessor:

____

____

____

© 2017 Knowledge E. All rights reserved. This workbook is intended for non-commercial use and may not be reproduced, distributed, or transmitted without the prior written permission of the publisher.

Interactive session

Giving feedback

Watch this video about a CVS history

Your Notes:

-
-
-
-
-
-
-
-
-

© 2017 Knowledge E. All rights reserved. This workbook is intended for non-commercial use and may not be reproduced, distributed, or transmitted without the prior written permission of the publisher.

Developing and running OSCE – common pitfalls

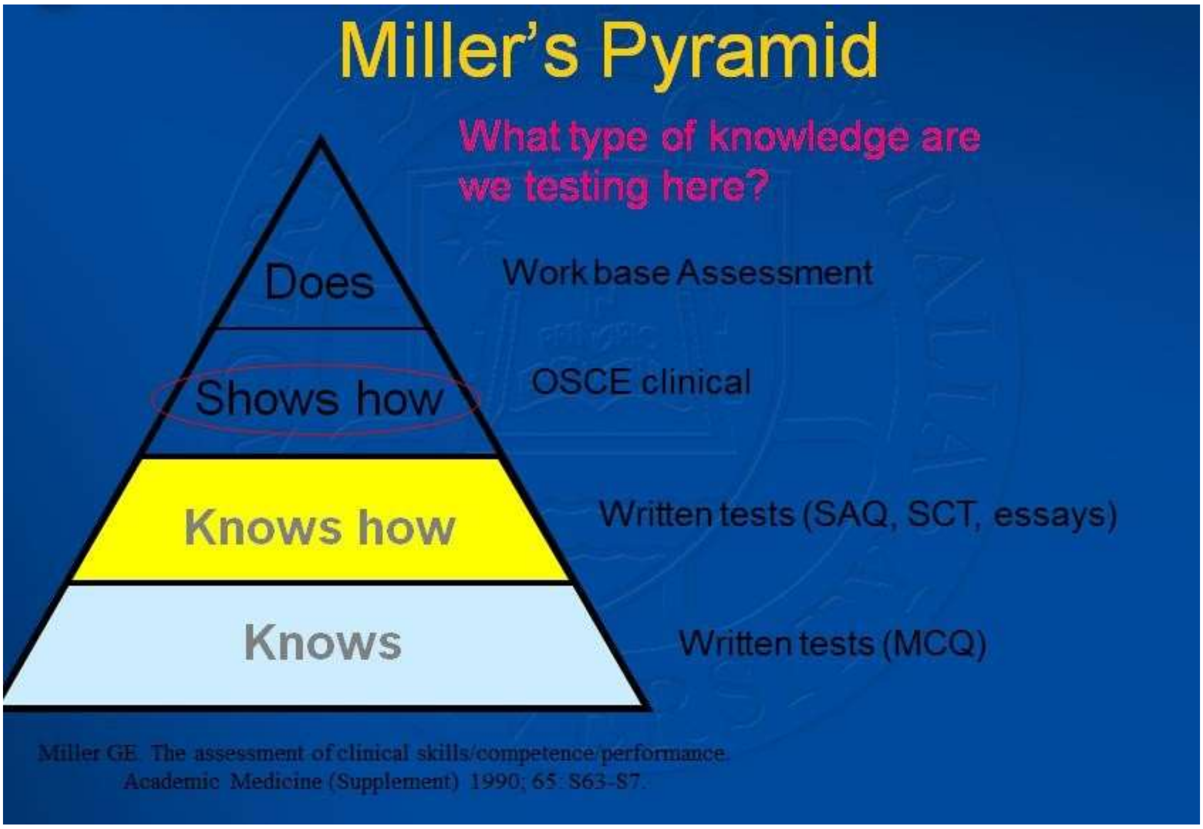

What do we want to assess?

Students be able to show us:

- Taking a focused history
- Performing a targeted examination
- Competency in procedural skills
- Communication skills
- Clinical reasoning
- Clinical decision making
- Professionalism

© 2017 Knowledge E. All rights reserved. This workbook is intended for non-commercial use and may not be reproduced, distributed, or transmitted without the prior written permission of the publisher.

Pre-clinical years

- Systematic history taking of major systems (e.g. CVS, GIT, Resp,...)
- Systematic examination of major systems
- Procedural skills on plastic models
- Communication

Clinical years

- Focused history on common presentations (various disciplines)
- Targeted examination of relevant systems
- Procedural skills on plastic models
- Communication skills
- Professionalism

Marking guide:

Pre-clinical:

- Check box or ticking the box format

Clinical:

- Competency based
- Global scoring
- 'Fail', 'Borderline', 'Clear Pass', 'Excellent'

© 2017 Knowledge E. All rights reserved. This workbook is intended for non-commercial use and may not be reproduced, distributed, or transmitted without the prior written permission of the publisher.

OSCE Exam Blue Printing

• Determine the spread of topics to be assessed

• Weighting according to discipline?

• Identify areas to be assessed

Planning the OSCE

• Venue, date

• Examiners, actors, patients arrangement

• Training sessions

• Preparation for exam: signage, station details, marking forms

Before the OSCE Exam:

• Confirm attendance of all examiners

• Backup examiners available

• Confirm attendance of all actors

• Backup actors available

• Confirm attendance of all patients

• Backup patients available

• Backup plastic models or instruments available

• Sick bay for patients

Before the OSCE Exam:

• Walk the circuit

• Check signage

• Check station numbers

• Check student instruction of each station match content and marking form inside room

• Check timer

© 2017 Knowledge E. All rights reserved. This workbook is intended for non-commercial use and may not be reproduced, distributed, or transmitted without the prior written permission of the publisher.

Before Exam (examiners):

- Switch off mobile phone
- Wear name badge
- Check sign with real patient
- Check history or sign with actors
- Check instruments for exam

Contingency:

- Examiner, actor or patient not available, reserve/backup to step in
- Extra spare marking sheets in the folder for examiners
- Equipment not working, ask staff to replace with spare
- OSCE exam in-charge, should be around to troubleshoot

After the Exam

- Collect feedback from examiners, actors, patients to improve the assessment process
- Review the performance of students in each station
- Debriefing for examiners
- Thank the examiners, actors and patients

Standardisation of Examiners (13)

- Improve exam consistency and reliability
- Fairness to students
- Ensure similar standard of assessment
- Training session before the OSCE
- Briefing on the run-down of the OSCE exam
- Explanation of the format of marking
- Discussion on the scoring system
- Familiarisation with the individual station content and assessment details

© 2017 Knowledge E. All rights reserved. This workbook is intended for non-commercial use and may not be reproduced, distributed, or transmitted without the prior written permission of the publisher.

To do:

• Follow the instructions in the examiners guide

• Follow the wording and sequence of each question to be asked

• Check timing according to the instructions

• If student is completely off the track: prompt:

"Would you like to read the student information sheet again?"

• If there are more than one answer to the question, prompt: "anything else, any other differential diagnoses / investigations...."

Not to do:

• Ask additional questions in the station

• Giving hint or guide to the students

• Giving marks to signs that students do not actually elicited

• Commenting on students' performance: e.g. "Good", "excellent", "this is wrong", "You are hopeless"

• Giving additional marks to students you know well that 'should' be a 'distinction' student

Standardisation of Actors

• Improve exam consistency and reliability

• Fairness to students

• Ensure similar standard of assessment

• Training session before the OSCE

• Briefing on the run-down of the OSCE exam

• Ensure consistency in giving a history

• Ensure consistency in mimicking the 'signs'

• Need to check with the examiner on the day of exam

© 2017 Knowledge E. All rights reserved. This workbook is intended for non-commercial use and may not be reproduced, distributed, or transmitted without the prior written permission of the publisher.

To do:

• Follow the instructions in the actor guide

• Follow the details given in the history sheet

• Follow the described 'signs' to act out accordingly

• If being asked of questions that they don't know, say "normal" or "okay", or refer to the examiner for a response

Not to do:

• Make up extra history

• Make up signs

• Giving hint or guide to the students

• Doing additional manoeuvres when not instructed to do so

• Respond to medical terms while being asked for the history, e.g. haemoptysis, dysphagia

© 2017 Knowledge E. All rights reserved. This workbook is intended for non-commercial use and may not be reproduced, distributed, or transmitted without the prior written permission of the publisher.

Challenges in assessment

Assessing Professionalism: (14) (15)

- Professionalism is highly context specific
- Most clinical behaviour not observed by colleagues

Ginsburg, et al., 2000

Some solutions

- Focus upon behaviours, not traits
- Increase awareness for the need to collect many samples of performance
- Use of portfolios to encourage self-reflection
- Workplace based assessment with constructive feedback (Mini-CEX)
- Multiple assess points, multiple assessors

**Skill category**	
**Doctor-patient relationship skills**	**Item**
1.	Listened actively to patient
2.	Showed interest in patient as a person
3.	Recognized and met patient needs
4.	Extended him/herself to meet patient needs
5.	Ensured continuity of patient care
6.	Advocated on behalf of a patient
11.	Maintained appropriate boundaries
**Reflective skills**	
7.	Demonstrated awareness of limitations
8.	Admitted errors/omissions
9.	Solicited feedback
10.	Accepted feedback
12.	Maintained composure in a difficult situation
**Time management**	
14.	Was on time
15.	Completed tasks in a reliable fashion
17.	Was available to colleagues
**Interprofessional relationship skills**	
11.	Maintained appropriate boundaries
13.	Maintained appropriate appearance
16.	Addressed own gaps in knowledge and skills
18.	Demonstrated respect for colleagues
19.	Avoided derogatory language
20.	Maintained patient confidentiality
21.	Used health resources appropriately

© 2017 Knowledge E. All rights reserved. This workbook is intended for non-commercial use and may not be reproduced, distributed, or transmitted without the prior written permission of the publisher.

Explicit and transparent assessment rubrics/policy

Assessment rubrics:

- Available in student handbook
- Electronic copies on student portal at the beginning of the year
- Clinical Teachers and assessors trained and informed on marking standard & guidelines

Review and Quality Assurance

- Expert review on current assessment practice and process in the School
- Allow comparison with international accepted criteria and guidelines
- Commendation on area of excellence
- Identification of gaps and weakness in the Assessment program
- Recommendations for improvement
- External Review
- Accreditation Council visits: Assessment Standards
- Benchmarking with national and international Assessment collaborations

Benchmarking (Medical Schools & National Collaborations)

Purpose of benchmarking:

- Sharing assessment items - ↓ workload
- Developing a common assessment framework
- Enhancing and expanding discussion relation to common assessment
- Development and distribution of institution-level and student-level benchmark reporting (identified or anonymous)
- Engaging and training academics in developing quality assessment items – faculty development
- Collaborative assessment and evaluation of learning outcomes
- Identifying gaps in curriculum or teaching related to learning outcomes

Current SOMS benchmarking examples – (University of Notre Dame, Sydney):

- SOMF, University of Adelaide, UNSW, Flinders University (MCQ, SCT)
- Australian Medical School Assessment Collaboration (AMSAC) – end of Pre-clinical years
- Australian Medical Assessment Collaboration (AMAC) – (end of clinical years) graduating
- ACCLAiM – benchmarking OSCE style exam
- Australian Medical Council (AMC) – compare with International medical graduates (IMGs)

© 2017 Knowledge E. All rights reserved. This workbook is intended for non-commercial use and may not be reproduced, distributed, or transmitted without the prior written permission of the publisher.

Outcome of Benchmarking

- Developing and sharing high quality assessment items
- Ensure comparable standards of assessment across schools
- Ensure comparable students performances/standards across schools and with IMGs
- Enhance collaborations in assessment among schools
- Facilitate research and quality improvement in medical education

© 2017 Knowledge E. All rights reserved. This workbook is intended for non-commercial use and may not be reproduced, distributed, or transmitted without the prior written permission of the publisher.

Wrapping up- future collaborations

References:

1. Harden RM, Laidlaw JM. Essential Skills for a Medical Teacher: Elsevier Health Sciences UK; 2012.

2. DeVon HA, Block ME, Moyle-Wright P, Ernst DM, Hayden SJ, Lazzara DJ, et al. A Psychometric Toolbox for Testing Validity and Reliability. Journal of Nursing Scholarship. 2007;39(2):155-64.

3. Al-Eraky M, Marei H. A fresh look at Miller's pyramid: assessment at the 'Is' and 'Do' levels. Medical Education. 2016;50(12):1253-7.

4. Cilliers F, Van Schalkwyk SC, Tan CPL, Bezuidenhout J. Mapping undergraduate exit-level assessment in a medical programme : a blueprint for clinical competence? : research. African Journal of Health Professions Education. 2016;8(1):45-9.

5. Wan M. Using the script concordance test to assess clinical reasoning skills in undergraduate and postgraduate medicine. Hong Kong Medical Journal. 2015;21(5).

6. Nouh T, Boutros M, Gagnon R, Reid S, Leslie K, Pace D, et al. The script concordance test as a measure of clinical reasoning: A national validation study. American Journal of Surgery. 2012;203(4):530-4.

7. Humbert AJ, Miech EJ. Measuring Gains in the Clinical Reasoning of Medical Students: Longitudinal Results From a School-Wide Script Concordance Test. Academic Medicine. 2014;89(7):1046-50.

8. Lambert C, Gagnon R, Nguyen D, Charlin B. The script concordance test in radiation oncology: Validation study of a new tool to assess clinical reasoning. Radiation Oncology. 2009;4(1):7-.

9. Gagnon R, Charlin B, Lambert C, Carrière B, Van Der Vleuten C. Script concordance testing: More cases or more questions? Advances in Health Sciences Education. 2009;14(3):367-75.

10. Wan SH, Duggan P, Tor E, Hudson JN. Association between candidate total scores and response pattern in script concordance testing of medical students. Focus on Health Professional Education: A Multi-disciplinary Journal. 2017;18(2):26-35.

11. Thomas JD, Arnold RM. Giving Feedback. Journal of Palliative Medicine. 2011;14(2):233-9.

12. Kurtz S, Silverman J, Benson J, Draper J. Marrying content and process in clinical method teaching: enhancing the Calgary-Cambridge guides. Academic medicine : journal of the Association of American Medical Colleges. 2003;78(8):802-9.

13. Reid K, Smallwood D, Collins M, Sutherland R, Dodds A. Taking OSCE examiner training on the road: reaching the masses. Medical Education Online. 2016;21(1):32389-5.

© 2017 Knowledge E. All rights reserved. This workbook is intended for non-commercial use and may not be reproduced, distributed, or transmitted without the prior written permission of the publisher.

14. Cruess RL, Cruess SR, Steinert Y. Amending Miller's Pyramid to Include Professional Identity Formation. Academic medicine : journal of the Association of American Medical Colleges U6 - ctx_ver=Z3988-2004&ctx_enc=info%3Aofi%2Fenc%3AUTF-8&rfr_id=info%3Asid%2Fsummonserialssolutionscom&rft_val_fmt=info%3Aofi%2Ffmt%3Akev%3Amtx%3Ajournal&rftgenre=article&rftatitle=Amending+Miller%27s+Pyramid+to+Include+Professional+Identity+Formation&rftjtitle=Academic+medicine+%3A+journal+of+the+Association+of+American+Medical+Colleges&rftau=Cruess%2C+Richard+L&rftau=Cruess%2C+Sylvia+R&rftau=Steinert%2C+Yvonne&rftdate=2016-02-01&rfteissn=1938-808X&rftvolume=91&rftissue=2&rftspage=180&rft_id=info%3Apmid%2F26332429&rftexternalDocID=26332429&paramdict=en-US U7 - Journal Article. 2016;91(2):180.

15. Hodges BD, Ginsburg S, Cruess R, Cruess S, Delport R, Hafferty F, et al. Assessment of professionalism: Recommendations from the Ottawa 2010 Conference. Medical Teacher. 2011;33(5):354-63.

© 2017 Knowledge E. All rights reserved. This workbook is intended for non-commercial use and may not be reproduced, distributed, or transmitted without the prior written permission of the publisher.
