# Supplementary material for: Radical reform of the undergraduate medical education program in a developing country: the Egyptian experience
Source: BMC Med Educ. 2023 Mar 3;23:143. doi: 10.1186/s12909-023-04098-3 (PMC9983512; doi:10.1186/s12909-023-04098-3)
Supplement: Supplementary file 4 — Additional file 4. Student survey 2018-2019. [file 12909_2023_4098_MOESM4_ESM.pdf]

Annex 4

Bachelor Degree New Medical Integrated Program

Student Survey 2018-2019

Faculty	
year of the study	
GPA	

	1.Stongly agree	2.agree	3. Neutral	4. Disagree	5. Extremely disagree
1. Faculty web are helpful for teaching and learning					
2. Faculty members are aware of modules and methods of teaching.					
3. Faculty members have good communication skills.					
4. Awareness week was helpful to the student.					
5. Study guide is helpful to the students.					
6. Time schedule are suitable for to study					
7. Teaching load in the lectures is suitable.					
8. There are methods for the students, other than lectures, to communicate with the faculty members.					
9. There is a need for external courses to study medicine					
