# Supplementary material for: Radical reform of the undergraduate medical education program in a developing country: the Egyptian experience
Source: BMC Med Educ. 2023 Mar 3;23:143. doi: 10.1186/s12909-023-04098-3 (PMC9983512; doi:10.1186/s12909-023-04098-3)
Supplement: Supplementary file 5 — Additional file 5. Student survey 2019-2020. [file 12909_2023_4098_MOESM5_ESM.pdf]

Annex 5

New Medical Integrated Program for the Bachelor Degree

Student Survey 2019-2020

Faculty	
year of the study	
GPA	

	1.Stongly agree	2.agree	3. Neutral	4. Disagree	5. Extremely disagree
1. Learning outcomes are explained at each lectures					
2. Faculty web are easy and helpful					
3. Teaching elective courses is useful to me					
4. Teaching research skills is mandatory in the curriculum.					
5. Teaching clinical skills occurs early in the curriculum.					
6. I get exposed to research ethics skills during the teaching process.					
During the era of Covid 19:					
7. Teaching practical lessons through online is effective					
8. Teaching clinical lessons through online is effective					
9. Small group learning through online is effective					
10. Practical lesson through online is more effective than face to face.					
11. Overall, I prefer face to face than online.					
12. I am satisfied with online teaching.					
13. I am satisfied with the online formative assessment.					
14. I am satisfied with the online continuous assessment.					
15. I am satisfied with the					

**online final assessment**					
