# Supplementary material for: Radical reform of the undergraduate medical education program in a developing country: the Egyptian experience
Source: BMC Med Educ. 2023 Mar 3;23:143. doi: 10.1186/s12909-023-04098-3 (PMC9983512; doi:10.1186/s12909-023-04098-3)
Supplement: Supplementary file 6 — Additional file 6. Faculty perceptions and practices in the new (Integrated) program questionnaire. [file 12909_2023_4098_MOESM6_ESM.pdf]

Annex 6

Faculty Perceptions and Practices in the New (Integrated) Program Questionnaire

University Name	
Email	
Department	
Title	
Number of taught courses	

	Yes	No	I don't Know
1. My institute introduces an Orientation Session for the students in the beginning of the program			
2. There is a curriculum management team			
3. I participate in a module/course management team			
4. The number of working Faculty is suitable for the implementation of the integrated program			
5. The students divided in small groups in the theoretical sessions			
6. There is a study guide for each course/module			
7. Basic sciences laboratories equipment is adequate to qualify the students with the required skills			
8. Phase I students are trained in the clinical skills laboratory			
9. The integrated systems qualify the students with ability to relate the basic knowledge to the clinical practice			
10. The integrated programs includes Self Directed Learning activities			
11. The integrated program students utilize the Egyptian Knowledge Bank (EKB) resources			
12. The students' feedback about the course/module is regularly obtained			
13. Students Formative Assessments are used during the course/module			
14. The practical/clinical examinations are integrated			
15. . The examinations are objective (Objective Questions e.g. MCQs , Modified Essays)			
16. The practical/clinical examinations are objective (OSPE/OSCE)			
17. The examinations are designed according to the Exam Blueprint			
18. In my institute, Logbook / Porto folio, is one of students' assessment methods			
19. My institute has an active questions bank			
20. The required topic titles and assessment tool (Checklist) were announced electronically			
21. The review articles were electronically submitted by the			

students			
22. The review articles submitted by the students were enough for students evaluation			
	Yes	No	Partially/som-etimes
23. The learning resources are accessible for the students			
24. The Faculty use the modern technology for teaching in the integrated program			
25. The integrated program enhances the interaction between the faculty from different departments			
26. I use the Learning Management System (LMS) to communicate with the students			
27. I use other electronic methods to communicate with the students *			
28. The students interact effectively during online synchronous sessions			
	Yes		No
29. I attended faculty development workshops about ONLINE teaching and learning methods and assessment methods in the integrated program?			
30. There is integration between the face to face and the online components of the modules/courses			
31. The number of students is suitable for the implementation of the integrated program			
32. I clarify the learning outcomes (LOs) to the students in the beginning of the lecture			
33. There is internet in my institute			
34. My institute used the online examinations for students' evaluation during COVID-19 pandemic			
35. My institute used online Summative assessment			
36. I think online examinations were enough for students' evaluation			
37. My institute used the electronically submitted review articles to evaluate the students during COVID-19 pandemic			

- What are your suggestions to improve the Blended Learning?
- What are your suggestions to improve the implementation of the integrated program?
- What are the required faculty development programs?
